# Sequence basis of Barnacle Cement Nanostructure is Defined by Proteins with Silk Homology

**DOI:** 10.1038/srep36219

**Published:** 2016-11-08

**Authors:** Christopher R. So, Kenan P. Fears, Dagmar H. Leary, Jenifer M. Scancella, Zheng Wang, Jinny L. Liu, Beatriz Orihuela, Dan Rittschof, Christopher M. Spillmann, Kathryn J. Wahl

**Affiliations:** 1 Chemistry Division, Code 6176, US Naval Research Laboratory, 4555 Overlook Ave, SW, Washington, DC, USA; 2 Center for Biomolecular Science and Engineering, Code 6900, US Naval Research Laboratory, 4555 Overlook Ave, SW, Washington, DC, USA; 3 Nicholas School of the Environment and Earth Sciences, Duke University Marine Laboratory, 135 Duke Marine Lab Rd, Beaufort, NC, USA

## Abstract

Barnacles adhere by producing a mixture of cement proteins (CPs) that organize into a permanently bonded layer displayed as nanoscale fibers. These cement proteins share no homology with any other marine adhesives, and a common sequence-basis that defines how nanostructures function as adhesives remains undiscovered. Here we demonstrate that a significant unidentified portion of acorn barnacle cement is comprised of low complexity proteins; they are organized into repetitive sequence blocks and found to maintain homology to silk motifs. Proteomic analysis of aggregate bands from PAGE gels reveal an abundance of Gly/Ala/Ser/Thr repeats exemplified by a prominent, previously unidentified, 43 kDa protein in the solubilized adhesive. Low complexity regions found throughout the cement proteome, as well as multiple lysyl oxidases and peroxidases, establish homology with silk-associated materials such as fibroin, silk gum sericin, and pyriform spidroins from spider silk. Distinct primary structures defined by homologous domains shed light on how barnacles use low complexity in nanofibers to enable adhesion, and serves as a starting point for unraveling the molecular architecture of a robust and unique class of adhesive nanostructures.

Natural materials have remarkable functional properties and ambient processability: threads with high strength, glues that cure underwater, and ceramics that resist fracture. Multiple functions emerge when materials evolve under harsh conditions, for instance, many natural marine glues exhibit added bond toughness to withstand dynamic ocean currents or wave action at the seashore. For sessile marine organisms such as barnacles, oysters and tubeworms, the adhesive is required to function for a lifetime and persists long afterwards. Such permanent adhesives use inventive adaptations to achieve durability, such as hierarchical structuring and chemical modification at the molecular and supramolecular level^1^,^2^. Despite inspiring scientific inquiry for more than a century^3^ and impeding maritime operations even today^4^, the permanent bond of adult barnacles is among the least understood. In contrast to marine organisms that use glues to fabricate protective shelters (e.g., sand-castle worm tubes^5^, case-maker fly larva retreats^2^, and amphipod tubes^6^,^7^) or tie themselves to rocks, (e.g., mussel byssus threads^8^,^9^) adult barnacles produce their adhesive interface in a sequential process hidden under their base as a part of their normal growth cycle^10^,^11^. The recent finding that barnacle adhesive is nanostructured and held together as an amyloid-like material^12^,^13^,^14^ further distinguishes it from archetypal marine adhesives processed into solid foams^1^,^9^,^15^ or spun threads^2^,^6^.

Barnacle glue maintains a high beta sheet content^12^,^16^. The adhesive nanofibers produced are particularly insoluble and consist of numerous protein components^17^,^18^. Over a 20 year period, five protein sequences have been identified through the use of aqueous denaturants such as guanidine hydrochloride, formic acid, and urea^17^,^18^,^19^. These treatments have solubilized a fraction of barnacle cement, though the inability to target the primary interactions between cement proteins remains a significant roadblock. Of the known proteins, putative functions have been assigned to each component based on the sequence chemistries and their observed abundance upon disassociation. For example, a 19 kDa hydrophilic component is assigned as a versatile surface-binding protein while 52 and 100 kDa proteins are thought to comprise the bulk of fibrillar cement^18^,^20^,^21^. High levels of aliphatic residues in these cement components have led to a hypothesis that the barnacle adhesive is partially held together and operates through a hydrophobic effect^17^,^20^,^22^,^23^,^24^. However, it has been difficult to establish the main components of bulk cement as many demonstrate *in vitro* fibril formation^14^,^25^,^26^,^27^, while a significant portion remains insoluble and, therefore, unidentifiable.

Progress in understanding cement has also left inconsistencies in knowledge of protein composition and the resulting chemical interactions that hold nanofibrils together. An observed release of proteins from bulk glue in the presence of reducing agents led to the assertion that disulfide bonding plays a role in cement cross-linking^19^,^23^,^28^. However, there is no spectroscopic evidence for S-S bonding in the cement layer^29^,^30^ and cysteine content remains low or nonexistent in the proteins thought to comprise bulk glue or the native cement itself^10^,^19^. Discrepancies exist between the handful of identified components and the amino acid composition of the proteinaceous cement layer, while major components remain unsequenced. Many uncertainties arise from the use of techniques poorly suited to sequence and analyze mixed protein samples or aggregates.

Recently, extensive studies of mRNA and protein expression in barnacles have provided systemic insight into metamorphosis, adult development and molting^31^,^32^,^33^,^34^. This has been enabled by the development of high throughput RNA sequencing (RNA-seq) as well as tandem mass spectrometry methods that together create proteomic databases directly from complex materials^35^. These methods are ideal to study the composition of barnacle glue, as protein complexes are sequenced as cleaved peptide fragments. Analysis of transcript sequences collected from the basal membranes of barnacles *A. amphitrite* and *T. japonica* confirmed cement proteins are produced in tissues contained just above the baseplate^34^,^36^,^37^. Both studies performed on *A. amphitrite* have revealed two additional cement proteins that share primary structure and amino acid chemistry with existing sequences, suggesting that barnacles produce cement proteins in specialized subfamilies^34^,^37^. However, transcriptomic studies have revealed few new proteins in addition to those identified more than a decade ago. Without direct proteomic sequencing of solubilized cement, insight into nanostructure composition and the relationship of components with themselves or other adhesive materials remains a challenge.

To determine the sequence basis of cement nanostructure, we have established a comprehensive barnacle cement proteome. In this work, we employ a targeted approach to disassemble the adhesive interface and report *ca*. 50 proteins, most of which were previously unidentified. We construct our proteome using three strategies: i) milligram-scale collection of cement attached by barnacles onto glass microspheres and collection of a thick and opaque cement type, ii) non-covalent breakdown of collected materials through the use of organic solvents and iii) transcriptome-led protein sequencing of individual bands from SDS PAGE gels as well as proteins collected from the entire gel lane. Based on the findings from these collections, two classes of proteins are defined, glycine/serine-rich cement proteins (GSrCPs) and leucine-rich cement proteins (LrCPs). The former share a conserved primary structure with previously identified proteins. Polar GSrCPs are found to share homology to certain silk motifs through short domains that define a distinct primary structure. The collection techniques and subsequent data analysis offer the clearest and most comprehensive picture of barnacle cement composition to date and reveal a prominent role for GSrCPs and low complexity in the construction of barnacle cement nanofibrils.

## Results

### Attachment Surfaces are Coated with Dense Nanofibrils Dissolved by Organic Solvents

**Figure 1:**
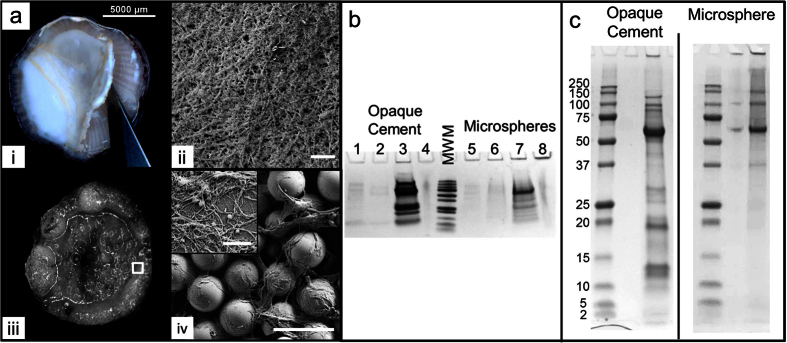
Collection and breakdown of cement samples from adult *A. amphitrite* barnacles. (**a**) Examples of fine nanofibrils networked into dense materials observed in multiple collection methods: (i) Thick and opaque cement layer secreted from the barnacle underside, (ii) SEM micrographs of (i) showing a mat consisting of fine nanomaterials, scale bar represents 1 μm. (iii) Underside of a barnacle settled on a bed of glass microspheres after one day, with large accumulations at the periphery, (iv) SEM micrographs of barnacle-adhered microspheres entrapped by cement secretions, scale bar is 100 μm. Inset, 2.25 μm^2^ image showing close up of nanofibrils on beads, scale bar is 500 nm. (**b**) Breakdown of collected cement using ethyl acetate (lanes 1 and 4), urea (lanes 2 and 6), hexofluoroisopropanol (lanes 3 and 7), and dithiothreitol (lanes 4 and 8) solvents, eluted proteins analyzed by SDS-PAGE and (**c**) Full length PAGE run of ‘opaque’ and ‘microsphere’ adhesives after dissolution by HFIP with an abundant protein released at 63 kDa among other well-known bands at 250, 100, 35, and 19 kDa. Left is ‘opaque’ cement, right is ‘microsphere’. Uncropped whole gels from (**b,c**) are available in Supplementary Figure S7.

The adhesive secretions of barnacles are particularly challenging to access – the proteinaceous layer is typically about a micron in thickness and is bonded permanently during the life of the barnacle to the substrate; in Amphibalinid species this layer is protected under an opaque calcium carbonate base plate. Thus, three collections (Fig. 1a) were compared: (a) material from large barnacles (5–10 mm) removed from silicone panels that exhibited an opaque, fibrillar adhesive (‘opaque’ – Fig. 1a,i,ii); (b) material on glass microspheres (48–85 μm diameter) that remained attached to barnacles grown on and then lifted off a bead bed (‘bead’, Fig. 1a iii,iv); and (c) adhesive secreted under barnacles grown for two months on sodium aluminoborate glass that forms a hydrated reaction layer facilitating barnacle removal, referred to as a ‘medallion’ collection^34^,^38^. Adhesive from ‘opaque’ and ‘bead’ collections were not readily soluble in known protein denaturants such as Urea (Fig. 1b) and a moderately polar organic solvent ethyl acetate (EtAc), but readily released protein in strongly polar hexafluoroisopropanol (HFIP). Like the thicker fibrillar adhesive under the barnacle, fibrils on beads were insoluble in EtAc and Urea, where materials were observed to remain until exposure to HFIP (Supplementary Figure S1)^13^,^30^. Solubilizing the adhesive with HFIP left little residual material in the loading well of PAGE gels and yielded a distribution of protein bands with similar molecular weights as those found previously by others^17^,^19^. Generally, breakdown using HFIP yields 5 main bands (150, 100, 63, 35, 20 kDa) from ‘bead’ collections and 7 bands (250, 100, 63, 35, 20, 19, 14 kDa) from ‘opaque’ adhesive. The ‘medallion’ samples were sufficiently solubilized by DTT, with bands at (250, 70, 63, 20 kDa). In contrast to previous studies, the most prominent band in these solubilized samples were at an apparent molecular mass of 63 kDa.

### MS/MS Analysis of Solubilized Nanofibrils Yields Many Proteins and Reveals the Sequence of a Major Cement Component

**Figure 2:**
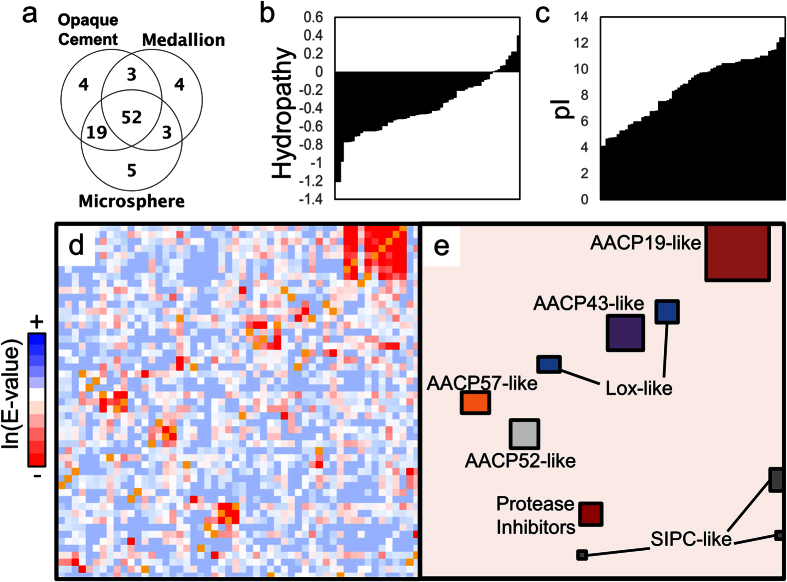
Broad sequence properties of the combined proteome showing (**a**) Number of identified proteins shared among sample collection methods that contain at least four peptides. (**b**) Bar graph of proteins sorted by GRAVY values showing 80% are hydrophilic with hydropathy values at around −0.4, while 14% are more hydrophobic with values above 0. (**c**) Graph of isoelectric point (pI) values in increasing order, with an average of 8.9±2.4 where 40% of the proteins lie above 10. (**d**) Pairwise E-values for 52 protein sequences represented by a two toned look up table, clustered into 7 regions of homologous proteins with 10 outlying individual pairs. Darker blue regions have no homology. Yellow squares indicate identity. (**e**) Outline and identification of self-similar protein families, named by the highest scoring protein sequence.

Combining peptide sequences derived from whole lane sequencing, defined as aggregate bands, of ‘opaque,’ ‘bead,’ and ‘medallion’ collections reveals the barnacle adhesive interface is chemically complex and develops from a large number of proteins. A total of 1113 unique peptides belonging to 90 putative proteins were identified by searching a translated cDNA database generated from the cement gland region as reported in our previous transcriptomic study^34^. Comparative analysis of proteins from aggregate MS/MS data demonstrates a set of common proteins among collection methods that share at least four unique peptide matches (Fig. 2a). More than two-thirds of the proteins were identified in all three collections, while nearly 90% of identified proteins were shared when only comparing ‘opaque’ and ‘bead’ collections (Fig. 2a). Inspection of all peptides identified from three types of collected cement shows that a majority are shared among 25 proteins coded by our transcript database (Supplementary Table S1). Identified proteins exist largely as mildly hydrophilic, while a small portion have Grand Average Hydropathy (GRAVY) values above 0 (Fig. 2b). All collection methods reveal the presence of previously reported *Amphibalanus amphitrite* cement proteins (AaCPs, where the theoretical molecular weight in kDa is appended to the name) AaCP19^34^, AaCP100^36^, AaCP114^34^, AaCP52^36^, and settlement inducing complex (SIPC) AaCP170, but not AaCP14.

The most abundant proteins separated by SDS-PAGE were found at 63 kDa. This band, as well as other prominent bands, were excised from gels and digested with trypsin for MS/MS analysis. Peptides derived from the 63 kDa band were found to be coded by transcript comp41238_c0_seq1 from our transcriptome database, containing 448 amino acids with a predicted molecular weight of 43 kDa, named by previous convention as AaCP43 (Supplementary Table S2). In most cases, AaCP43 maintained the highest number of peptides among single 63 kDa band analysis (Supplementary Table S2) and typically occupied one of the highest peptide counts in the aggregate band analysis (Supplementary Table S1). Although this protein runs at 63 kDa by PAGE, analyzed peptides from aggregate and isolated bands cover only the established coding region of 43 kDa, leaving a 20 kDa discrepancy that could be due to protein complexation or other post-translational modifications. To shed light on this, the 43 kDa coding region was recombinantly expressed in *E. coli* and found to behave similarly as the Wild-Type protein, migrating to *ca*. 60 kDa by SDS-PAGE (Supplementary Figure S2 and Supplementary Methods). Treatment of the solubilized glue with glycosidases also yielded no shift in molecular weight of this band, suggesting that heavy glycosylation and protein complexation are not responsible for the 20 kDa shift. Acid hydrolysis of this band matches the sequence composition for AaCP43 (Supplementary Figure S3) and also is consistent with the composition of major, but unsequenced, bands in other barnacle species including the major 58 kDa band found by Naldrett, *et. al* in *B. eburneus*^17^ and a 68 kDa band identified by Kamino, *et. al* in *M. rosa*^19^. Compositions of these bands across species share greater than 10% glycine, serine, threonine and alanine, indicating that homologs of AaCP43 may exist in at least two other species. The full translated sequence length of AaCP43 was verified by RT-PCR on collected mRNA samples^34^, revealing only three nucleotide mismatches from the RNA-seq data (Supplementary Figure S4).

### Cement Proteome Contains a High Number of Homologous Proteins

Sequences from aggregate band analysis of ‘opaque,’ ‘beads’, and ‘medallion’ samples were pooled with single band analysis, including an additional sample of barnacles removed from glass coverslips^34^ (Supplementary Tables S3–5) to produce a combined cement proteome. Sequence similarity among highest scoring proteins was explored by performing pairwise alignment (BLAST), where expectation values (e-values) between 52 selected proteins were clustered hierarchically by shared homologies into a 2D array (Fig. 2d). Identical positions are marked in yellow, regions of similarity (e-value <10^−4^) are red, and blue gradients represent low and null values with no homology as the darkest blue value. A number of high similarity regions emerge along with several discrete points outside these regions (Fig. 2e, listed in Table 1). The largest region labeled ‘19-like’ is centered around AaCP19 which comprises nine proteins- five full length sequences with MW ranging from 19 to 49 kDa, three partial C-/N-terminus sequences, and one more partial sequence we found only in the translated protein database. These sequences are grouped by a family name ‘Aa19’. Two other regions are found around barnacle proteins AaCP52 and SIPC^36^, as well as one around the abundant protein AaCP43 discussed above (grouped by family names ‘Aa52’ and ‘Aa43’ respectively). We found three additional smaller clusters. The first of these clusters consists of three full protein sequences having no known homology to previously reported proteins; this was labeled ‘AaCP57-like’ (grouped by family name ‘Aa57’) using the predicted MW of the highest scoring protein sequence. The last two larger groupings were identified by searching the nrNCBI protein database, revealing homology to protease inhibitors and the enzyme lysyl oxidase (Lox), labeled ‘Lox-like’ (see Table 2). Finally, discrete points of similarity revealed several other pairs including two 105 kDa proteins (Table 1). Other known homologues (AaCP100/AaCP114^34^ and AaCP20-1/AaCP20-2^37^) were found to verify the similarity matrix.

**Figure 3:**
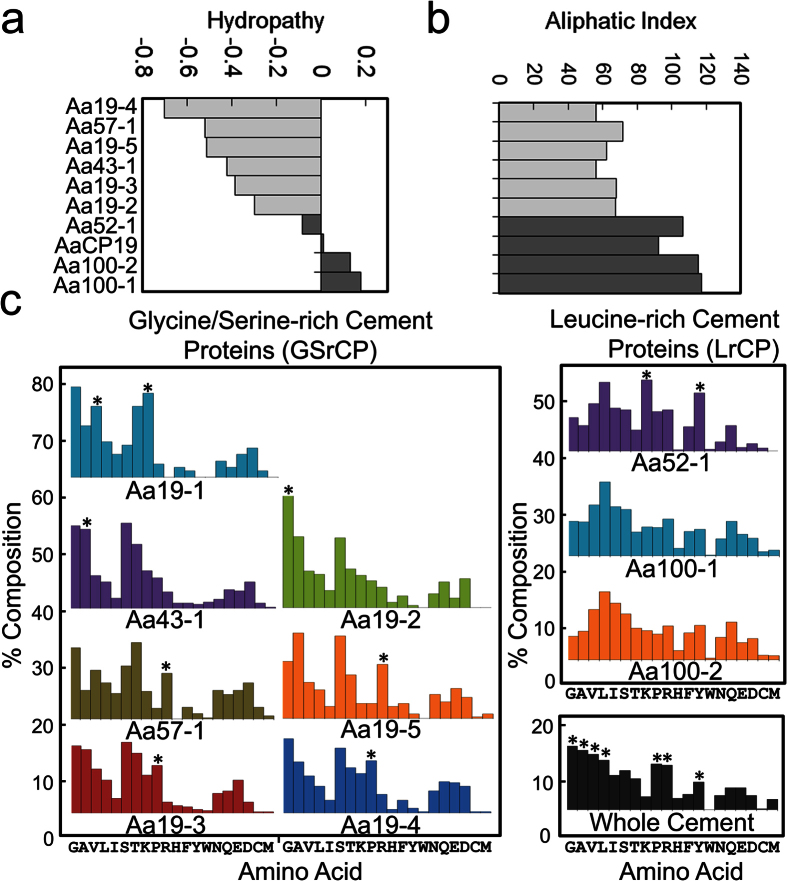
Classification of homologous cement proteins by amino acid composition. (**a**) Corresponding values of hydropathy ranging from hydrophobic to hydrophilic, (**b**) Aliphatic index values of cement proteins are bimodal, when relative volumes of alanine, valine, isoleucine and leucine side chains are compared. (**c**) Left, polar GSrCPs defined by an abundance of glycine, alanine, serine and threonine residues. Right, aliphatic LrCPs defined by an abundance of valine, isoleucine and leucine residues and higher aliphatic index values. Bottom right, acid hydrolysis of whole cement from microspheres yields an abundance of glycine, alanine, serine and threonine in addition to hydrophobic residues. Asterisks indicate residues that are abundant in individual components.

The amino acid composition of previously named proteins AaCP19, AaCP52, AaCP100 and AaCP114 lie at a hydrophobic extremity (*ca*. −0.1 to 0.2) while the new Aa43, Aa57 and Aa19 families are closer to the average hydropathy for the cement proteome shown in Fig. 2b (*ca*. −0.4). This distinction is more pronounced (Fig. 3b) when a measure of alanine, valine, isoleucine and leucine side chain volume, the aliphatic index of the proteins, is considered. Therefore, we categorize proteins by composition into Glycine/Serine-rich cement proteins (GSrCP, including the Aa19, Aa43, and Aa57 families) and Leucine-rich cement proteins (LrCP, including the Aa52 and Aa100 families) as defined in Table 1 and Fig. 3c. The GSrCPs are polar with an abundance of Gly/Ala/Ser/Thr residues while LrCPs are aliphatic, rich in Val/Leu/Ile residues. We propose a new naming scheme for the two broad categories of cement proteins found, GSrCP-[family name]-[x] for new polar proteins and LrCP-[family name]-[x] for existing aliphatic proteins. In addition to broad compositional similarities, various single residues are enhanced within each class of proteins, marked by asterisks in Fig. 3c to include Arg/Pro/Lys/Tyr. For comparison the compositional profile of hydrolyzed ‘bead’ collection is shown in the lower right corner of Fig. 3c.

### GSrCPs Share Conserved Primary Structure with Archetypal Cement Proteins

**Figure 4:**
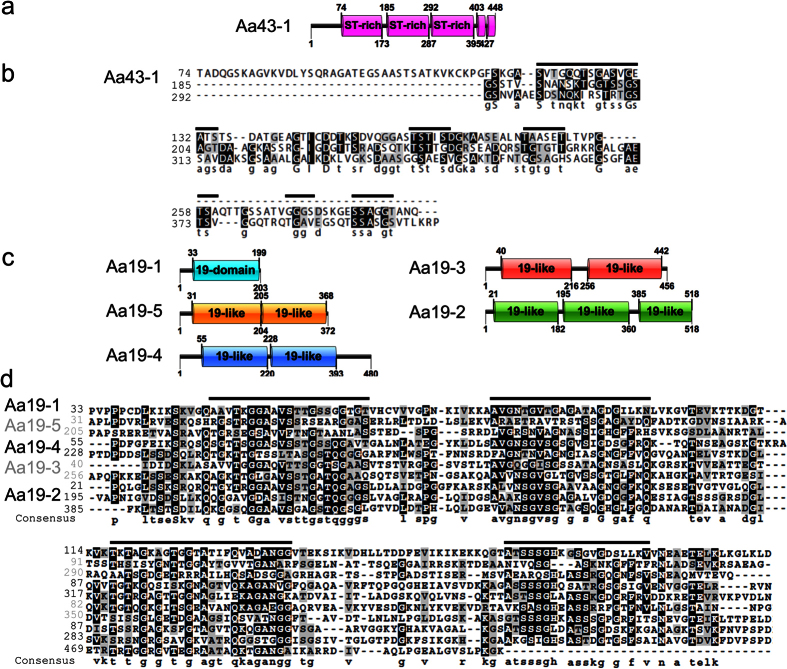
Sequence homology of Aa43 and Aa19 GSrCPs. (**a**) Primary structure of Aa43-1 showing three homologous domains spanning *ca*. 100 residues each. (**b**) Multiple sequence alignment of three low complexity domains in Aa43-1 showing conservation of 60% residues with shared chemistry, (**c**) primary structure of four GSrCPs with repeated domains of high homology to AaCP19 (**d**) Multiple sequence alignment of all 19-homologous domains, where conserved regions are composed mainly of low complexity residues. Black lines highlight regions of low complexity.

The Aa19 family shares considerable primary structure homology with other known cement proteins as revealed by multiple sequence alignment analysis. A 166 residue domain from AaCP19 is highly conserved among Aa19-2, -3, -4 and -5 proteins. While Aa19-3, -4, and -5 each contain two repeated blocks, Aa19-2 is comprised of three repeated blocks of AaCP19. Multiple alignment of these 10 domains is shown in Fig. 4d, where 102 residues out of 170 (60%) are conserved among at least half of the protein sequence. Residues present at 10% or above (Ala/Gly/Ser/Thr) match the distinct amino acids found in the GSrCPs, with the addition of Val. The high number of alignments among the Aa19 family reveals that low complexity regions are conserved, marked by black lines in Fig. 4b,d, consisting of small and uncharged side-chains. Inspection of the GSrCP sequence Aa43-1 yields a triple-repeat segment of *ca*. 110 residues, with 63 conserved (58%, Fig. 4a,b). In both protein families, each domain maintains a pattern of low complexity, further flanked by regions with highly charged residues (e.g. Lys, Arg, Glu, Asp, Gln, and Asn).

### Cement contains Multiple Oxidases and Proteases

22 proteins coded by our transcript ID database share significant homology to sequenced proteins in the non-redundant NCBI database (nrNCBI). These proteins are summarized in Table 2, where seven are in the oxoreductase family and include three lysyl oxidases (AaLox-1, -2, -3) and three peroxinectins (AaPxt-1, -2, -3) and one peroxidase (AaPx-1). The *A. amphitrite* Lox is closely related to a homolog found in *Drosophila melanogaster* (Dmloxl-2, CAB99481), containing two cysteine-rich scavenger domains as well as the lysine tyrosylquinone co-factor linkage, copper binding site and conserved cytokine receptor-like region^39^ (Supplementary Figure S5). AaPxt-1 and -2 found are homologous to a heme-peroxidase found in the fly larvae of *Hesperophylax occidentalis*^40^ (KM384736, Supplementary Figure S6), with three distal and two proximal heme cavities. Additionally, we find both a serine protease (AaSP) and three homologous protease inhibitors (AaPI-1, -2, -3) in the proteome. Three forms of MULTIFUNCin are identified in *A. amphitrite* (AaMulti-1, -2, -3), which are cues that promote predation as well as cyprid settlement^41^.

### New Cement Protein Sequences Display Silk-like Signatures

**Figure 5:**
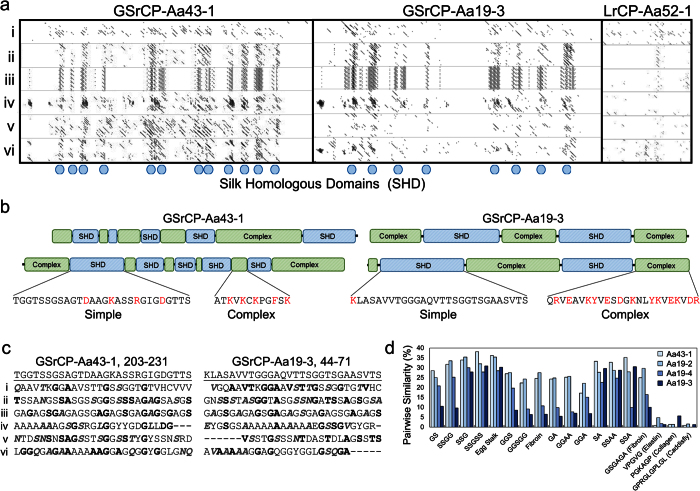
Shared primary structure of polar GSrCPs. (**a**) Recursive dot plot analysis of GSrCP-Aa43-1, GSrCP-Aa19-3, and LrCP-Aa52-1 against archetypal silk proteins, highlighting regions of low complexity along primary sequences. (i) AaCP19, (ii) Egg Stalk Silk from *M. signata*, (iii) Heavy Chain Fibroin from *B. mori*, (iv) Heavy Chain Fibroin from *R. fugax*, (v) Sericin I from *B. mori*, (vi) Spidroin I from *N. clavipes*. Dots represent silk homologous domains (SHDs). (**b**) Distinct alternating primary structure observed in GSrCPs defined by alternating silk homologous and complex domains. (**c**) Pairwise alignment of archetypal silk proteins to representative SHDs, demarked by dot plotting, where bold letters are identical to the query sequence and italicized bold letters are chemically similar. (**d**) Stringency of primary sequence in GSrCP low complexity regions measured by pairwise alignment to *de novo* silk-like motifs, showing higher stringency with [SS] based models over [GG]. Percentages are the number of similar and identical alignments between pairs, divided by cement protein sequence length.

To explore the homology of polar GSrCP sequences with other organisms, we remove compositional bias filtering from BLAST to search nrNCBI using only the native scoring matrix (BLOSUM62). Searches limited to Arthropods (including Crustaceans) by this method yield a considerable number (200+) of additional alignments. While aliphatic LrCPs maintain alignments solely among barnacles as seen in filtered nrNCBI searches, polar GSrCPs share significant homology to large numbers of non-barnacle proteins (summarized in Table 3, full results in Supplementary Table S6). Unfiltered nrNCBI searching corroborates multiple sequence analysis from Fig. 4d, where the highest scoring results for the Aa19 family belong to AaCP19 from *A. amphitrite* and other barnacle species^8^. However, certain Aa19 proteins, such as Aa19-2 and -5, exhibit greater similarities with fiber-forming proteins over AaCP19 (Table 3). Aa43-1, on the other hand, exhibits strong alignment with multiple silk protein constructs including fibroins, egg stalks, and gum sericins. In fact, silk-related proteins are common among the highest scoring alignments across a majority of GSrCPs. Proteins appearing with highest frequency are a moth egg stalk silk (ACN87362) as well as the silk gum sericin (AGN03940.1), occupying the top two alignments for Aa43-1, Aa19-2 and Aa19-5 as well as other proteins coded from the transcript database (comp27593_c0_seq1, comp27343_c0_seq1, comp48220_c0_seq1). Interestingly, some polar GSrCPs include significant homologies to portions of spider silk sequences: Aa19-2 with orb weaver dragline silk (AAL32375.1) and minor ampullate spidroins, while Aa19-3 aligns with a pyriform spidroin (ADK92884.1) used to adhere dragline silks to solid surfaces. Alignment of silk protein sequences to GSrCPs visualized by dot plotting (Fig. 5a) reveals that silk homology is confined into short domains, defining a distinct primary structure in this class of cement proteins (Fig. 5b). Regions between silk homologous domains display a high number of basic residues rich in arginine and lysine, highlighted in Fig. 5b as ‘complex’.

Since GSrCPs favor alignment with sequences that contain repetitive -SS- or S-X motifs, we ask whether the observed low complexity requires a degree of order. Pairwise alignments between GSrCPs and both natural (fibroin, elastin, collagen) and *de novo* fiber-forming motifs (-GS-, -SSGG-, -SSG-, -SSSSG-, -GGS-, -GGGGS-, -GA-, -GGAA-, -GGA-, -SA-, -SSAA-, -SSA-) spanning the length of cement proteins show a clear preference for motifs containing -SSXX-. For certain GSrCPs (Aa19-2 and -3), di-ser sequences are highly favored over di-gly and di-ala motifs. Previous alignments with the nrNCBI protein database are supported by this analysis, where lacewing stalk silk rich in -SSSS- motifs are preferred over the alternating motif of fibroin from *B. mori* (-GSGAGA-). In contrast, alignments are weak with other well known proteinaceous fibers that contain gly-based motifs such as elastin and collagen. G-X and S-X dipeptide motifs define either crystalline sheet or amorphous coil regions of fibroin-based silk fibers depending on the polymorphism of X^42^,^43^. G-X and S-X content in GSrCPs are loosely conserved compared to silks^42^,^44^,^45^,^46^, typically polymorphic between Ala/Ser and to a lesser extent Arg/Lys/Asx/Glx, and does not seem to obey the Pauling-Corey anti-parallel model for fibroin^45^. Glycine percentages in these domains remain below 40%, also indicating they have a propensity for fibril formation but not for the elastic silk-like properties^47^.

## Discussion

Integrated proteomic and transcriptomic analysis reveals that nanofibrillar cement in barnacle adhesive is comprised primarily of a new and unique family of polar proteins. These proteins are expressed specifically in the adhesive plaque; they were identified using mRNA sequences derived from sub-mantle tissues where cement glands and other cement proteins are found^34^. GSrCPs were not found in translated mRNA libraries assembled from other regions of the organism (main body, side plates^48^), or in MS/MS analysis of fluid collected from canals that line the side plates. Furthermore, both filtered and unfiltered searches against all arthropod nrNCBI sequences yield no homologies to known cuticle proteins, which are in intimate contact with the cement^13^.

Fibers are the dominant ultrastructure in barnacle adhesive. Cement collections display a matted, nanofibrillar ultrastructure^12^,^13^,^49^,^50^,^51^, that forms in both sealed and porous attachment conditions, and is readily dissolved by polar solvents used to solubilize biomaterials rich in hydrogen bonds and β-sheet structures^52^,^53^,^54^. Relative abundance of proteins in MS/MS data confirms PAGE observations, where Aa43 and polar GSrCPs occupy a significant fraction of cement. In fact, whole solubilized samples consist of 30–50% identifiable peptides that belong to GSrCPs, with protein abundance index (emPAI) values 5–10 times greater than those of aliphatic LrCP components thought to comprise bulk cement (Table 1). Studies of cement from other species show that homologous proteins are released after exposure to aqueous denaturants, but not in abundance as seen when using HFIP^17^,^18^,^19^. Our findings collectively demonstrate that GSrCPs are a major component of nanofibril ultrastructures, held together by dense hydrogen bonding and released by highly polar solvents.

Many primary sequences throughout the polar GSrCPs are conserved, typically rich in glycine, serine, threonine and alanine residues. These proteins are generally unrelated to previous cement components by composition and sequence, however many contain repeated blocks aligned with a well-studied 19 kDa cement protein^21^. Thus, low complexity regions are widely found throughout cement, defining new and chemically unique sequences while relating them to previously identified cement proteins. Low complexity is commonly associated with fibrous materials, ranging from ordered structures (i.e. silks, elastin, and keratin) to disordered domains that become pathological amyloids^55^. In amyloid formation, glycine-rich domains organize into liquid droplets, a precursor state for long range beta sheet formation^55^. In cement, silk-homologous proteins encompass the largest cohort in our proteome; 22 sequences maintain e-values with silks ranging from 10^−10^–10^−33^ while they occupy *ca*. 30% of all identifiable peptides in cement, where most align to a specific egg stalk silk. Since GSrCPs comprise a large portion of a fibrous material with amyloid-like secondary structure^12^,^14^, we believe homology with arthropod silks to be biologically significant. The highly varied compositions of other proteins in the barnacle cement proteome such as LrCPs, SIPCs and other miscellaneous enzymes indicate that alignments are not from a bias in overall barnacle protein composition.

Aquatic arthropods such as caddisfly larvae^2^,^56^ and certain amphipods^6^,^7^ have recently been found to employ silks as mortar to construct their protective housing underwater. Caddisfly (e.g., *H. consimilus* and *H. occidentalis*) belong to the more evolutionary distant insects while amphipods (e.g., *C. bonellii*) are closer neighbors, divergent from barnacles by four ancestors^57^. These findings underscore the evidence that crustaceans can share adhesive traits with distantly related insects. At least one GSrCP was found homologous to multiple pyriform spidroins, a nanofibrillar cement material used by orb-weaving spiders to attach dragline silk to a solid substrate^58^. Unlike spun silks, dragline cement is secreted as a viscous fluid that cures over time^58^,^59^, a process more in line with observations of barnacle adhesive viscosity^60^,^61^ and interface development^49^,^62^. Barnacles do not appear to draw fibers from ductwork to deliver adhesion chemistries, rather, the adhesive is more likely shaped through spontaneous protein folding similar to amyloid formation. Additionally, many GSrCPs align with sericin^63^, a sticky silk gum protein that binds fibers together and to various surfaces. Barnacle adhesives may have evolved homologous properties with these silk-associated cements, which are examples of the first materials identified with shared sequence, ultrastructure and function.

Finally, our results show that barnacle adhesives likely undergo chemical processing subsequent to their self-assembly. Cross-referencing the cement proteome with nrNCBI reveals multiple enzymes within the adhesive collections. Of particular interest are peroxinectins, which have been implicated in catalyzing oxidative crosslinking of caddisworm silk^64^ and lysyl oxidases, which acts to modify lysine side chains and participates in crosslinking collagen, elastin fibrils as well as cuticular tissues^39^. The high number of lysines in exposed complex domains of GSrCPs suggests that they would be available for cross-linking. Phosphorylation of the abundant serine and threonine residues in GSrCPs presents another possible modification, as has been identified in many aquatic^2^,^65^ adhesives including secretions from cyprids and adult barnacles^66^,^67^. Indeed, kinases were identified in transcriptome sequencing of the cement gland region^34^. However, our analysis of the solubilized cement did not reveal these entries or any other kinases. Previous infrared spectroscopy of the adhesive from underneath live barnacles and reflected from the top of demineralized cement plaques have shown little evidence for organophosphate bonds, although x-ray photoelectron spectroscopy of newly deposited adhesive revealed the presence of a small amount of phosphate^13^,^30^. Abundant basic residues found throughout the cement proteome (40% of the proteins with average pI above 10) could serve to aid barnacle adhesion; for example, lysines and arginines have recently been found essential in displacing saltwater cations to allow direct interaction with surface oxides^16^,^68^. The identification of relationships to other silk-producing arthropods and functional molecular chemistries offers a first glimpse into how barnacle cement is constructed and functions. Future work includes identifying what role the enzymes may play, the extent of post-translational modifications to GSrCPs, and how these materials fit into the curing mechanism of the cement.

## Methods

### Informatics analysis of adhesive proteome

Informatics software used in this work: Gepard 1.4 with a BLOSUM62 scoring matrix (University of Vienna, Austria) using a window of 9 for dot plot analysis (displaying equal regions of the histogram), Cluster 3.0 (University of Tokyo, Japan) for hierarchical clustering of e-values (Distances measured by uncentered Pearson correlation, clustered using mean linkages), TreeView 3.0 (Stanford, USA) to generate 2D correlated e-values in Fig. 2d and Table 1. BLASTP (NIH, USA) 2.2.32+ was used for annotation of proteome entries, using a BLOSUM62 scoring matrix with gap penalties as 11 for existence and 1 for extension. RADAR (EMBL-EBI, UK) was used for self sequence alignment, Clustal Omega 1.2.1 (EMBL-EBI, UK) was used for multiple sequence alignment and EMBOSS Needle (EMBL-EBI, UK) with gap open penalty of 50 was used for pairwise alignment of silk-like segments identified by dot plots from Fig. 5. EMBOSS Water (EMBL-EBI, UK) with gap open penalty of 20 was used to find local alignments with *de novo* silk-like motif sequences to keep percentage of gaps below 5%. Boxshade 3.21 used to display alignment files with a 0.5 threshold of homology or identity for shading. IBS 1.0 (Sun Yat-sen University, China) was used to generate protein schema.

For sequence assignment of MS/MS data, assembled cDNA generated from RNA-seq performed previously^34^ were translated into a FASTA database (1044910 total entries) in six open reading frames with the EMBOSS transeq command (EMBL-EBI, UK). All identified transcript sequences from PAGE samples are then combined into a subset FASTA database and uploaded to BLASTP for pairwise alignment and e-value analysis. The natural log of all pairwise e-values are then submitted to Cluster 3.0 and plotted using a two-tone look up table with Treeview. Clusters with 10^−4^ or lower e-values are identified as a family and entered into Table 1. Grand average of hydropathicity (GRAVY) values were established using the system defined by Kyte and Doolittle^69^. Aliphatic index values were determined using methods defined by Ikai^70^. GRAVY and Aliphatic index values were calculated using ProtParam (SIB, Switzerland) software.

To find all possible cement proteins, the full transcriptome was searched for additional entries using the two domains identified in the 43-like and 19-like protein families using a hidden Markov search. Two additional sequences were found belonging to the 43-like family and one with homology to AaCP19 were found, making a total of six 43-like proteins and nine 19-like proteins. When this motif was used to search the broader NCBI database, only the previously identified 19 kDa proteins were retrieved.

### Barnacle Husbandry

*A. amphitrite* cyprids were settled on silicone-coated glass panels and reared at the Duke University Marine Laboratory as previously described^71^. Panels with adult barnacles were shipped to the Naval Research Laboratory (NRL) where they were maintained in an incubator operating at 23 °C with 12 h day/night cycles in 32 ppt artificial seawater (Instant Ocean, Blacksburg, VA). The barnacles were fed Artemia spp. nauplii (Brine Shrimp Direct, Ogden, UT) three times a week and the artificial seawater was changed once a week during which the algal growth was removed. Barnacles to be used for experiments were gently dislodged from the silicone-coated glass panels^13^, rinsed with distilled water, and placed on alternate substrates for the experiments.

### Cement Collection on Glass Microspheres

Barnacles were placed onto a bed of soda lime glass microspheres (48–85 μm) (Cospheric, Santa Barbara, CA) in ASW forming a bed of microspheres 2–3 mm in depth. After one week, barnacles were lifted off, and microspheres associated with their underside were gently transferred without damaging the barnacle. The microspheres were pooled from multiple animals (n = 6), rinsed 3 times in fresh D.I. water, and stored at 4–8 °C until use. Microspheres untouched by barnacles from the same dish were also collected and rinsed for background measurements.

### Collection of Opaque Adhesive

Adult barnacles that develop a thick white opaque adhesive (a.k.a., gummy glue) were gently removed from silicone panels and the ‘opaque’ glue was shaved or peeled off the baseplate without damaging the barnacle using an angled razor blade. ‘Opaque’ glue pieces were rinsed with D.I. water, pooled from multiple animals (n = 3) and placed in enough hexafluoroisopropanol (HFIP) to cover the pieces.

### Collection from Rinsed Plaques

Rinsed plaques were collected using previously developed methods^34^. Briefly, barnacles were transferred from silicone panels and settled on sodium aluminoborate (Na_2_O ∙ Al_2_O_3_ ∙ 3B_2_O_3_) glass substrates, which form a hydrated reaction layer (<25 μm thick) in aqueous environments that is resistant to barnacle adhesion^38^. After 8 weeks, barnacle bodies and side plates were carefully removed, leaving the base of the barnacles attached to the substrates which were then cleaned with a cotton swab in deionized water to remove loosely bound organic matter. Base plates were then demineralized by immersing substrates in 0.1 M ethylenediaminetetraacetic acid (EDTA) at room temperature for 48–72 h. Barnacle “medallions,” consisting of the cuticular layer and underlying barnacle secretions, were gently rinsed with deionized water then peeled off the aluminoborate glass substrates and pooled (n = 3) in 50 μL of Laemli sample buffer containing 300 mM dithiothritol (DTT).

### SDS-PAGE

‘Microsphere’ and ‘opaque’ samples were prepared by adding two bed volumes of HFIP to the microspheres and opaque glue collections and then sonicated in a water bath for 1 hour at room temperature. The HFIP was removed and evaporated to dryness by vacuum centrifuge. Next, for all three sample types, 25 μL of Laemmli sample buffer containing 300 mM DTT and 25 μL of D.I. water were added and incubated for 15 min at 95 °C.

The samples were separated by SDS-PAGE at 200 V constant on Any kD Mini-PROTEAN TGX precast gels (Bio-Rad, Hercules, CA) using Tris-SDS running buffer (25 mM Tris, 192 mM glycine, and 0.1% SDS, pH 8.3). Gels were then stained with either Bio-Safe Coomassie Stain (Bio-Rad, Hercules, CA) or Imperial protein stain (Thermo Fisher Scientific) for visualization.

### Amino Acid Analysis

Microsphere cement samples were subjected to acid hydrolysis using 6N aqueous HCl in a vacuum hydrolysis tube heated to 150 °C for 1.5 hours. Phenol was added to suppress the halogenation of tyrosine residues and thioglycolic acid to suppress cysteine oxidation. For quantification of amino acids, hydrolyzed samples were transferred to 6 mm wide borosilicate culture tubes, dried down by vacuum centrifugation and resuspended in 2:2:1 volumes of EtOH:H_2_O:Triethylamine (TEA). Samples were dried again and derivitized with phenylisothiocyanate (PITC) in a 7:1:1:1 solution (EtOH:H_2_O:PITC:TEA) for 30 mins at RT and dried. Analytical HPLC was carried out on an Agilent Infinity 1260 at 300 μL/min using a C18 4.6 × 150 mm Poroshell 120 column (2.7 μm bead-size, Agilent) heated to 38 °C, monitoring eluent absorbance at 250 nm. Solvent A was a 30 mM sodium acetate (pH 5.5) solution; Solvent B was 18 mM sodium acetate in a 70/30 acetonitrile/water mixture (pH 3.8), using an elution gradient by Smith *et al*.^72^. Elution times for each amino acid were established by first running PITC derivitized amino acid Standard H (Pierce Scientific).

Amino acid analysis of isolated 63 kDa PAGE band was performed by dissolving roughly 1 mg (hydrated mass) of ‘opaque’ glue in HFIP, dried by centri-vac, resuspended in sample buffer, and running 100 μL onto a 7 cm single lane gradient gel (Any kD TGX, Bio-Rad, Hercules, CA). Gel was stained using bio-safe coomassie (Bio-Rad, Hercules, CA) and immediately transferred to a 7 × 8.4 cm PVDF membrane (Sequi-Blot, Bio-Rad, Hercules, CA) using 10 mM CAPS running buffer (10% methanol, pH 11.0, vacuum degassed and filtered) under constant 100 mA current for 1.5 hrs. A bright band at 63 kDa was cropped by razor blade and sent to Bio-Synthesis Inc. (Lewisville, TX) for amino acid analysis. PVDF bands were hydrolyzed in 6N HCl for 24 hrs at 110 °C. Hydrolysate was dried and derivitized pre-column using 200 uL of aqueous 6-aminoquinolyl-N-hydroxysuccinimidyl carbamate (AQC) (AccQ-Tag, Waters) solution. 10 uL were injected onto a Waters Breeze 2 HPLC, where amino acid fluorescence and abundance were quantified by a Waters 2475 multi-λ fluorescence detector. Data shown in Supplementary Figure S3.

### PCR of GSrCP-Aa43-1

Total RNA from sub-mantle tissues was isolated as described previously^34^ and reverse transcribed by PCR to cDNA according to the manufacturer’s protocol (Life Technologies). The first strand cDNA was then used as a template along with the primers, AaCP43 F1 :

(5′ ATGCTGCCTGCCGCGATCC) and AaCP43 R1:

(5′ CTACCATTTAGGCTTATAC), to amplify the GSrCP-Aa43-1 transcripts by conducting PCR with the following steps: 95 °C for 30 sec, 56 °C for 1 min and 30 sec, 70 °C for 1 min and 30 sec, for 35 cycles. Resulting 1344 bp DNA fragments were isolated and then sequenced by using Sanger sequencing method (Eurofins MWG Operon; Louisville, KY). The assembled sequence was then aligned with the transcript comp41238_c0_seq1 using Clustal Omega (EMBL-EBI, UK). All RT-PCR related reagents and enzymes were purchased from Life Technologies (Carlsbad, CA) and reagents for DNA isolation were from Qiagen Inc (Valencia, CA).

### Tandem Mass Spectrometry and Sequence Assignment

Samples analyzed at NRL were processed as individual bands from protein extracts of each sample separated by SDS-PAGE, excised and digested in gel by trypsin. Peptides were extracted by 2% formic acid in 50/50 acetonitrile/water, followed by 100% acetonitrile. Digests were analyzed by liquid chromatography mass spectrometry/mass spectrometry (LC-MS/MS) using a Tempo-MDLC coupled to a TripleTOF 5600 mass spectrometer (AB Sciex, Foster City, CA). Tandem mass spectra were extracted by AB Sciex MS data convertor version 2. Aggregate samples were analyzed at the Texas A&M Protein Chemistry Lab (TAMU, College Station, TX) where protein extracts were run on SDS-PAGE for 10 minutes (see Fig. 1b), divided into four segments, digested by trypsin and extracted. Digests were analyzed by LC-MS/MS using a NanoLC 2-D (Eksigent, Dublin, CA) coupled to a LTQ Orbitrap Velos H/ETD (Thermo Scientific, Waltham, MA). Tandem mass spectra were extracted by Mascot Distiller (Matrix Science, London, UK) software. Two 62 kDa bands from ‘opaque’ and ‘microsphere’ cement samples were sent to BioProximity (BioP, Chantilly, VA) for multiple enzyme digestion and sequencing. Bands were cut into six pieces, where each piece was digested with a single enzyme: trypsin, chymotrypsin, Glu-C, alpha-lytic protease, pepsin and thermolysin. The six digestion products were then combined and injected for LC-MS/MS analysis. Digests were analyzed by LC-MS/MS using an Easy-nLC 1000 coupled to a Q Exactive Quadrupole-Orbitrap (ThermoFisher, Waltham, MA). Charge state deconvolution and deisotoping were not performed. All MS/MS samples were analyzed using Mascot (Matrix Science, London, UK; version 2.4.1) and X! Tandem (The GPM, thegpm.org; version CYCLONE (2010.12.01.1)). To translate assembled cDNA sequences generated from RNA-seq experiments^34^ into a searchable FASTA database, EMBOSS transeq command was used with 6 open reading frames. Mascot was set up to search the BarnALL_001 database (1045268 entries) assuming the digestion enzyme trypsin. X! Tandem was set up to search a subset of the BarnALL_001 database also assuming trypsin. Mascot and X! Tandem were searched with a fragment ion mass tolerance of 0.60 Da and a parent ion tolerance of 20 PPM for aggregate samples analyzed at TAMU. Samples analyzed at Bioproximity were searched with a fragment tolerance of 20 ppm and a parent ion tolerance of 0.8 Da. Samples analyzed at NRL were searched with a fragment tolerance of 0.2 Da and a parent ion tolerance of 0.2 Da. Deamidation of asparagine and glutamine, oxidation of methionine, acetylation of the n-terminus, and carbamidomethylation of cysteine were specified in Mascot as variable modifications. Glu- > pyro-Glu of the n-terminus, ammonia-loss of the n-terminus, gln- > pyro-Glu of the n-terminus, deamidation of asparagine and glutamine, oxidation of methionine, acetylation of the n-terminus, and carbamidomethylation of cysteine were specified in X! Tandem as variable modifications. Scaffold (version Scaffold_4.6.1, Proteome Software Inc., Portland, OR) was used to validate MS/MS based peptide and protein identifications. Peptide identifications were accepted if they could be established at greater than 80.0% probability by the Peptide Prophet algorithm^73^ with Scaffold delta-mass correction. Protein identifications were accepted if they could be established at greater than 95.0% probability and contained at least 4 identified peptides. Venn diagram in Fig. 2a was produced using a minimum of 4 identified peptides. Protein probabilities were assigned by the Protein Prophet algorithm^74^. Proteins that contained similar peptides and could not be differentiated based on MS/MS analysis alone were grouped to satisfy the principles of parsimony.

The mass spectrometry proteomics data have been deposited to the ProteomeXchange Consortium via the PRIDE^75^ partner repository with the dataset identifier PXD004293 and 10.6019/PXD004293.

## Additional Information

**How to cite this article**: So, C. R. *et al*. Sequence basis of Barnacle Cement Nanostructure is Defined by Proteins with Silk Homology. *Sci. Rep.* **6**, 36219; doi: 10.1038/srep36219 (2016).

**Publisher’s note**: Springer Nature remains neutral with regard to jurisdictional claims in published maps and institutional affiliations.

## Supplementary Material

Supplementary Information

## Figures and Tables

**Table 1:** Classification of cement-related proteins from aggregate sequencing into Glycine/Serine rich cement proteins (GSrCPs) and Leucine rich Cement Proteins (LrCPs).

Protein Name	Sequence Homology	PAGE MW	Protein MW	emPAI	pI	GRAVY	Aliphatic	State	Corresponding Transcript ID	ORF
Glycine/Serine rich Cement Proteins (GSrCP)										
**Aa43**										
Aa43-1	43	100, 66, 63, 58 AG	43460	17.87	9.73	−0.423	56.14	Full	comp41238_c0_seq1	4
Aa43-2	43	100, 67, 58, AG	28183	10.67	6.81	−0.47		Partial	comp27343_c0_seq1	4
Aa43-3	43	100, 63, 58, AG	20450	3.17	6.76	−0.167		Full	comp27593_c0_seq1	5
Aa43-4	43	63, 58, AG-MS	9710	—	6.39	0.219		Full	comp49909_c1_seq1	6
Aa43-5	43	Transcript	—	—	—	—	—	Partial	comp121192_c0_seq1	4
Aa43-6	43	63-MS	—	—	—	—	—	C-term	comp47087_c0_seq1	6
**Aa19**										
Aa19-1	19	19, AG	20154	53.75	10.45	0.013	92.17	Full	Aacp19k	
Aa19-2	19	63, AG-GG	49400	2.63	10.52	−0.3		Full	comp46137_c1_seq5	5
Aa19-3	19	250, 66, 63, AG	45320	1.36	9.52	−0.387	67.65	Full	comp40515_c0_seq1	5
Aa19-4	19	100, 66, 63, 58 AG	48610	0.82	8.4	−0.702	56.12	Full	comp45928_c1_seq1	6
Aa19-5	19	250, 63	38260	0.33	11.01	−0.515	62.34	Full	comp46330_c0_seq1	6
Aa19-6	19	63, AG-MS	9710	0.15	7.72	−0.459		C-term	comp33102_c0_seq1	4
Aa19-7	19	63, AG-GG	9620	0.74	10.77	−0.5		N-term	comp170921_c0_seq1	1
Aa19-8	19	63	8010	0.8	11.48	−0.511		N-term	comp87199_c0_seq1	5
Aa19-9	19	Transcript	—	—	—	—	—	C-term	comp49344_c0_seq1	2
**Aa57**										
Aa57-1	57	250, 100, 63	57120	2.26	8.74	−0.519	71.46	Full	comp47308_c0_seq1	4
Aa57-2	57		56710	0.16	9.15	−0.668		Full	comp46196_c0_seq1	5
Aa57-3	57	AG	59740	—	10.45	−0.768		Full	comp25334_c1_seq1	5
**Aa105**										
Aa105-1	105	250, 100, 66, 58, 63, 30	105009					Full	comp40644_c0_seq1	4
Aa105-2	105	100, GG	92610		8.88	−0.550		Full	comp39288_c0_seq1	5
Leucine rich Cement Proteins (LrCP)										
**Aa100**										
Aa100-1	100	100, AG	129500	4.77	10.25	0.178	117.08	Full	Aacp100k-1	
Aa100-2	100	100, AG	114220	1.71	10.01	0.133		Full	Aacp114k-2	
**Aa52**										
Aa52-1	52	Transcript	73010	0.19	11.02	−0.08		Full	Aacp52	
Aa52-2	52	AG	28070	—	10.75	−0.022		Full	comp33562_c1_seq1	4
Aa52-3	52	AG-MS	46260	3.83	10.28	−0.426		C-term	comp48220_c0_seq1	6

Amino acid sequences are coded by an open reading frame (ORF) identifier for protein MWs and sequence annotation, where ORF #s are appended to the Transcript ID in the proteome repository. Abbreviations are defined, AG: Aggregate, AG-MS: Aggregate microsphere, AG-GG: Aggregate gummy glue. emPAI values are all taken from one dataset of gummy MS/MS analysis for comparison.

**Table 2:** Annotations for cement proteins determined by searching nrNCBI.

Protein Name	NCBI Annotation	Accession	PAGE MW	Protein MW	E Value	Corresponding Transcript ID	ORF
AAmulti-1	MULTIFUNCin [Chthamalus fissus]	AFY13482.1	AG, 250	182231	0	comp48163_c0_seq1	4
AAmulti-2	MULTIFUNCin [Balanus glandula]	AFY13480.1	AG, 250	227597	0	comp39924_c0_seq1	5
AAmulti-3	MULTIFUNCin [Chthamalus fissus]	AFY13482.1		22835	3E-18	comp79394_c0_seq1	2
AAwap-1	Whey acidic protein protein 2 [Dufourea novaeangliae]	KZC10758.1	14	10903	4E-05	comp45426_c4_seq1	5
AAwap-2	Single whey acidic protein domain-containing protein isoform 2 [Penaeus monodon]	ACF28465.1 (XP_009052071.1)		22181	4E-15	comp32063_c0_seq1	4
AAsp	Neurotrypsin precursor [Mus musculus]	NP_032965.1	14, 63	16625	2E-10	comp44772_c1_seq1	2
AApxt-1	Peroxinectin [Pacifastacus leniusculus]	CAA62752.1 (EFX80390.1)	100, 58, 30, AG	62231	3E-48	comp45999_c0_seq1	4
AApxt-2	Peroxinectin [Eriocheir sinensis]	ADF87945.1 (XP_001946672.2)	58, AG	53067	2E-75	comp27704_c0_seq1	6
AApxt-3	Peroxinectin [Scylla serrata]	AAT12270.1 (XP_013775632.1)		15547	3E-22	comp83572_c0_seq1	4
AApxt-4	Chorion peroxidase [Daphnia magna]	KZS19290.1 (XP_015373617.1)		33033	3E-57	comp42709_c1_seq1	4
AAlox-1	Lysyl Oxidase-like 2 [Drosophila melanogaster]	CAB99481.1 (XP_001960248.1)	100, 63, AG	55870	2E-102	comp44772_c0_seq1	4
AAlox-2	Lysyl Oxidase-like protein [Stegodyphus mimosarum]	KFM62898.1	AG	9460	6.2E-2	comp81853_c0_seq1	6
AAlox-3	Lox2 [Drosophila busckii]	ALC40946.1 (EFX81056.1)		41383	1E-83	comp43852_c1_seq1	2
AApi-1	Protease inhibitor [Ctenocephalides felis]	AKR52931.1 (XP_014606419.1)	AG	42380	3E-44	comp44898_c1_seq1	4
AApi-2	Serpin B10 [Trachymyrmex septentrionalis]	KYN35588.1 (XP_011183022.1)	250, 66, 58	63948	2E-34	comp45011_c0_seq2	6
AApi-3	Protease inhibitor [Ctenocephalides felis]	AKR52931.1 (XP_013104691.1)	AG	44390	7E-44	comp44997_c0_seq1	6
AAmuc*	Intestinal mucin [Operophtera brumata]	KOB70769.1 (AGR65306.1)	63, 58, AG	46260	1E-85	comp43534_c0_seq1	4
AAtrx	Thioredoxin		AG		0	Thioredoxin	
AAsilk-1*	cross-beta structure silk protein 1 [Mallada signata]	ACN87361.1 (XP_012246550.1)		55051	3E-10	comp46330_c0_seq1	6
AAsilk-2*	cross-beta structure silk protein 1 [Mallada signata]	ACN87361.1		21890	2E-11	comp27593_c0_seq1	5
AAsilk-3*	cross-beta structure silk protein 1 [Mallada signata]	ACN87361.1 (CEP02176.1)		28200	7E-12	comp27343_c0_seq1	4
AAsilk-4*	cross-beta structure silk protein 2 [Mallada signata]	ACN87362.1 (XP_013410251.1)		54012	1E-17	comp48220_c0_seq1	6

ORF identifiers are appended to the Transcript ID in the proteome repository. Starred entries indicate results from unfiltered BLAST searching, without low complexity and compositional bias filters. Accession numbers in parentheses correspond to hypothetical proteins or proteins with predicted function that have higher e-values.

**Table 3:** Search results for GSrCPs against unfiltered nrNCBI, showing highest alignment with silk and cement proteins.

	Cement Homology	E value	Coverage	Silk Homology	E value	Coverage
Leucine rich Cement Proteins (LrCPs)						
Aa100-1	cement protein 100k [Amphibalanus amphitrite]	0	1	—	—	—
	114 kDa cement protein [Amphibalanus amphitrite]	0	0.84	—	—	—
	cement protein-100k [Megabalanus rosa]	0	0.84	—	—	—
Aa100-2	114 kDa cement protein [Amphibalanus amphitrite]	0	1	—	—	—
	cement protein 100k [Amphibalanus amphitrite]	0	0.98	—	—	—
	cement protein-100k [Megabalanus rosa]	0	1	—	—	—
Aa52-1	52 kDa cement protein [Amphibalanus amphitrite]	0	1	—	—	—
	52kDa cement protein [Megabalanus rosa]	2.00E-158	0.99	—	—	—
AaCP20-1	cement protein 20 kDa-1 [Amphibalanus amphitrite]	7.00E-87	0.92	—	—	—
	cement protein 20 kDa-3 [Amphibalanus amphitrite]	1.00E-40	0.84	—	—	—
	cement protein 20 kDa-2 [Amphibalanus amphitrite]	2.00E-27	0.91	—	—	—
Glycine/Serine rich Cement Proteins (GSrCPs)						
AaCP19	19 kDa cement protein [Amphibalanus amphitrite]	7.00E-141	1	—	—	—
	cement protein-19k [Fistulobalanus albicostatus]	2.00E-77	0.85	—	—	—
	cement protein-19k [Balanus improvisus]	3.00E-60	0.85	—	—	—
Aa19-2	cement protein-19k [Fistulobalanus albicostatus]	2.00E-20	0.86	cross-beta structure silk protein 1 [Mallada signata]	9.00E-32	0.98
	cement protein-19k [Megabalanus rosa]	4.00E-19	0.92	silk sericin MG-1 [Galleria mellonella]	7.00E-25	0.92
	19 kDa cement protein [Amphibalanus amphitrite]	8.00E-19	0.88	cross-beta structure silk protein 2 [Mallada signata]	8.00E-25	0.91
Aa19-3	cement protein-19k [Megabalanus rosa]	4.00E-29	0.81	pyriform spidroin 2 [Nephila clavipes]	5.00E-15	0.93
	cement protein-19k [Fistulobalanus albicostatus]	1.00E-27	0.74	piriform spidroin [Nephila clavipes]	3.00E-11	0.96
	19 kDa cement protein [Amphibalanus amphitrite]	3.00E-25	0.84	piriform-like spidroin [Nephilengys cruentata]	1.00E-10	0.86
Aa19-4	cement protein-19k [Fistulobalanus albicostatus]	1.00E-28	0.69	silk sericin MG-1 [Galleria mellonella]	2.00E-17	0.7
	19 kDa cement protein [Amphibalanus amphitrite]	2.00E-22	0.81	cross-beta structure silk protein 1 [Mallada signata]	1.00E-16	0.72
	cement protein-19k [Megabalanus rosa]	3.00E-20	0.81	cross-beta structure silk protein 2 [Mallada signata]	2.00E-14	0.73
Aa19-5	cement protein-19k [Balanus improvisus]	4.00E-08	0.69	cross-beta structure silk protein 1 [Mallada signata]	1.00E-10	0.95
	—	—	—	sericin 3 precursor [Bombyx mori]	1.00E-09	0.88
	—	—	—	cross-beta structure silk protein 2 [Mallada signata]	1.00E-09	0.94
Aa43-1	cement protein-19k [Balanus improvisus]	1.50E-01	0.26	cross-beta structure silk protein 1 [Mallada signata]	3.00E-33	0.8
	—	—	—	silk sericin MG-1 [Galleria mellonella]	7.00E-29	0.81
	—	—	—	cross-beta structure silk protein 2 [Mallada signata]	3.00E-26	0.8

## References

[b1] Stewart R. J., Weaver J. C., Morse D. E. & Waite J. H. The tube cement of *Phragmatopoma californica*: a solid foam. J. Exp. Biol. 207, 4727–4734 (2004). 15579565 10.1242/jeb.01330

[b2] Stewart R. J. & Wang C. S. Adaptation of caddisfly larval silks to aquatic habitats by phosphorylation of H-fibroin serines. Biomacromolecules 11, 969–974 (2010). 20196534 10.1021/bm901426d

[b3] Darwin C. A monograph on the sub-class Cirripedia, with figures of all the species. In Living Cirripedia, The Balanidae, (or sessile cirripedes); the Verrucidae Vol. 2 (The Ray Society, London, 1854).

[b4] Schultz M. P., Bendick J. A., Holm E. R. & Hertel W. M. Economic impact of biofouling on a naval surface ship. Biofouling 27, 87–98 (2011). 21161774 10.1080/08927014.2010.542809

[b5] Zhao H., Sun C., Stewart R. J. & Waite J. H. Cement proteins of the tube-building polychaete *Phragmatopoma californica*. J. Biol. Chem. 280, 42938–42944 (2005). 16227622 10.1074/jbc.M508457200

[b6] Kronenberger K., Dicko C. & Vollrath F. A novel marine silk. Naturwissenschaften 99, 3–10 (2012). 22057952 10.1007/s00114-011-0853-5

[b7] Kronenberger K., Moore P. G., Halcrow K. & Vollrath F. Spinning a marine silk for the purpose of tube-building. Journal of Crustacean Biology 32, 191–201 (2012).

[b8] Waite J. H. & Tanzer M. L. Polyphenolic substance of *Mytilus edulis* novel adhesive containing L-dopa and hydroxyproline. Science 212, 1038–1040 (1981). 17779975 10.1126/science.212.4498.1038

[b9] Waite J. H., Andersen N. H., Jewhurst S. & Sun C. Mussel adhesion: finding the tricks worth mimicking. J. Adhesion 81, 297–317 (2005).

[b10] Power A. M. . Mechanisms of adhesion in adult barnacles. Biological Adhesive Systems: From Nature to Technical and Medical Application (eds. von Byern J. & Grunwald I.) 153–168 (Springer, 2010).

[b11] Kamino K. Molecular design of barnacle cement in comparison with those of mussel and tubeworm. J. Adhesion 86, 96–110 (2010).

[b12] Barlow D. E. . Characterization of the adhesive plaque of the barnacle *Balanus amphitrite*: amyloid-like nanofibrils are a major component. Langmuir 26, 6549–6556 (2010). 20170114 10.1021/la9041309

[b13] Burden D. K. . Growth and development of the barnacle *Amphibalanus amphitrite:* time and spatially resolved structure and chemistry of the base plate. Biofouling 30, 799–812 (2014). 25115515 10.1080/08927014.2014.930736 PMC4159999

[b14] Nakano M. & Kamino K. Amyloid-like conformation and interaction for the self-assembly in barnacle underwater cement. Biochemistry 54, 826–835 (2015). 25537316 10.1021/bi500965f

[b15] Wang C. S., Svendsen K. K. & Stewart R. J. Morphology of the adhesive system in the sandcastle worm. In Biological Adhesive Systems: From Nature to Technical and Medical Application (eds. von Byern J. & Grunwald I.) 169–179 (Springer, 2010).

[b16] Stewart R. J., Ransom T. C. & Hlady V. Natural underwater adhesives. J. Polym. Sci. B Polym. Phys. 49, 757–771 (2011). 21643511 10.1002/polb.22256 PMC3104275

[b17] Naldrett M. J. & Kaplan D. L. Characterization of barnacle (*Balanus eburneus* and *B. cenatus*) adhesive proteins. Mar. Biol. 127, 629–635 (1997).

[b18] Kamino K., Odo S. & Maruyama T. Cement proteins of the acorn barnacle, Megabalanus rosa. Biol. Bull. 190, 403–409 (1996). 8679743 10.2307/1543033

[b19] Kamino K. . Barnacle cement proteins: Importance of disulfide bonds in their insolubility. J. Biol. Chem. 275, 27360–27365 (2000). 10840046 10.1074/jbc.M910363199

[b20] Kamino K. Mini-review: barnacle adhesives and adhesion. Biofouling 29, 735–749 (2013). 23802872 10.1080/08927014.2013.800863

[b21] Urushida Y. . Identification and functional characterization of a novel barnacle cement protein. FEBS J. 274, 4336–4346 (2007). 17683335 10.1111/j.1742-4658.2007.05965.x

[b22] Kamino K., Nakano M. & Kanai S. Significance of the conformation of building blocks in curing of barnacle underwater adhesive. FEBS J. 279, 1750–1760 (2012). 22404823 10.1111/j.1742-4658.2012.08552.x

[b23] Naldrett M. J. The importance of sulfur cross-links and hydrophobic interactions in the polymerization of barnacle cement. J. Mar. Biol. Assoc. UK 73, 689–702 (1993).

[b24] Jonker J. L. The natural adhesive of the goose barnacle *Lepas anatifera*: the functional morphology and chemistry of the adhesive, PhD Thesis, National University of Ireland, Galway (2013).

[b25] Nakano M., Shen J.-R. & Kamino K. Self-assembling peptide inspired by a barnacle underwater adhesive protein. Biomacromolecules 8, 1830–1835 (2007). 17518440 10.1021/bm0612236

[b26] Liang C., Li Y. Q., Liu Z. M., Wu W. J. & Hu B. R. Protein aggregation formed by recombinant cp19k homologue of *Balanus albicostatus* combined with an 18 kDa N-terminus encoded by pET-32a(+) plasmid having adhesion strength comparable to several commercial glues. Plos One 10, e0136493, doi: 10.1371/journal.pone.0136493 (2015). 26317205 PMC4552757

[b27] So C. R. . Self-assembly of protein nanofibrils orchestrates calcite step movement through selective nonchiral interactions. ACS Nano 9, 5782–5791 (2015). 25970003 10.1021/acsnano.5b01870

[b28] Mori Y., Urushida Y., Nakano M., Uchiyama S. & Kamino K. Calcite-specific coupling protein in barnacle underwater cement. FEBS J. 274, 6436–6446 (2007). 18021251 10.1111/j.1742-4658.2007.06161.x

[b29] Wiegemann M., Kowalik T. & Hartwig A. Noncovalent bonds are key mechanisms for the cohesion of barnacle (Balanus crenatus) adhesive proteins. Mar. Biol. 149, 241–246 (2006).

[b30] Barlow D. E., Dickinson G. H., Orihuela B., Rittschof D. & Wahl K. J. *In situ* ATR-FTIR characterization of primary cement interfaces of the barnacle *Balanus amphitrite*. Biofouling 25, 359–366 (2009). 19263278 10.1080/08927010902812009

[b31] Okazaki Y. & Shizuri Y. Structures of six cDNAs expressed specifically at cypris larvae of barnacles, *Balanus amphitrite*. Gene 250, 127–135 (2000). 10854786 10.1016/s0378-1119(00)00184-0

[b32] Chandramouli K. H. . Transcriptome and proteome dynamics in larvae of the barnacle *Balanus amphitrite* from the Red Sea. BMC Genomics 16, 1063, doi: 10.1186/s12864-015-2262-1 (2015). 26666348 PMC4678614

[b33] Bacchetti De Gregoris T. . Construction of an adult barnacle (*Balanus amphitrite*) cDNA library and selection of reference genes for quantitative RT-PCR studies. BMC Mol. Biol. 10, 62, doi: 10.1186/1471-2199-10-62 (2009). 19552808 PMC2713238

[b34] Wang Z. . Molt-dependent transcriptomic analysis of cement proteins in the barnacle *Amphibalanus amphitrite*. BMC Genomics 16, 859, doi: 10.1186/s12864-015-2076-1 (2015). 26496984 PMC4619306

[b35] Guerette P. A. . Accelerating the design of biomimetic materials by integrating RNA-seq with proteomics and materials science. Nat. Biotechnol. 31, 908–915 (2013). 24013196 10.1038/nbt.2671

[b36] Lin H. C. . First study on gene expression of cement proteins and potential adhesion-related genes of a membranous-based barnacle as revealed from Next-Generation Sequencing technology. Biofouling 30, 169–181 (2014). 24329402 10.1080/08927014.2013.853051

[b37] He L. S., Zhang G. & Qian P. Y. Characterization of two 20 kDa-cement protein (cp20k) homologues in Amphibalanus amphitrite. PLoS One 8, e64130, doi: 10.1371/journal.pone.0064130 (2013). 23717550 PMC3661472

[b38] Fears K. P., Scancella J. M., Orihuela B., Rittschof D. & Wahl K. J. Surface-active borate glasses as antifouling materials. Adv. Mater. Interfaces 2, 201500370, doi: 10.1002/admi.201500370 (2015).

[b39] Molnar J. . Drosophila lysyl oxidases Dmloxl-1 and Dmloxl-2 are differentially expressed and the active DmLOXL-1 influences gene expression and development. J. Biol. Chem. 280, 22977–22985 (2005). 15811848 10.1074/jbc.M503006200

[b40] Wang C. S., Pan H., Weerasekare G. M. & Stewart R. J. Peroxidase-catalysed interfacial adhesion of aquatic caddisworm silk. J. R. Soc. Interface 12, 112, doi: 10.1098/rsif.2015.0710 (2015). PMC4685843 26490632

[b41] Zimmer R. K. . A multifunctional chemical cue drives opposing demographic processes and structures ecological communities. Ecology 97, 2232–2239 (2016). 27859065 10.1002/ecy.1455 PMC5116919

[b42] Zhou C.-Z. . Silk fibroin structural implications of a remarkable amino acid sequence. PROTEINS: Structure, Function, and Genetics 44, 119–122 (2001). 10.1002/prot.1078 11391774

[b43] Wilson D., Valluzzi R. & Kaplan D. L. Conformational transitions in model silk peptides. Biophys. J. 78, 2690–2701 (2000). 10777765 10.1016/S0006-3495(00)76813-5 PMC1300858

[b44] Inoue S. . Silk fibroin of *Bombyx mori* is secreted, assembling a high molecular mass elementary unit consisting of H-chain, L-chain, and P25, with a 6:6:1 molar ratio. J. Biol. Chem. 275, 40517–40528 (2000). 10986287 10.1074/jbc.M006897200

[b45] Marsh R. E., Corey R. B. & Pauling L. An investigation of the structure of silk fibroin. Biochimica Et Biophysica Acta 16, 1–34 (1955). 14363226 10.1016/0006-3002(55)90178-5

[b46] Mita K., Ichimura S. & James T. C. Highly repetitive structure and its organization of the silk fibroin gene. J. Mol. Evol. 38, 583–592 (1994). 7916056 10.1007/BF00175878

[b47] Rauscher S., Baud S., Miao M., Keeley F. W. & Pomes R. Proline and glycine control protein self-organization into elastomeric or amyloid fibrils. Structure 14, 1667–1676 (2006). 17098192 10.1016/j.str.2006.09.008

[b48] Zhang G. . Chemical component and proteomic study of the *Amphibalanus* (=*Balanus*) *amphitrite* Shell. Plos One 10, e0133866, doi: 10.1371/journal.pone.0133866 (2015). 26222041 PMC4519255

[b49] Golden J. P. . Imaging active surface processes in barnacle adhesive interfaces. Langmuir 32, 541–550 (2016). 26681301 10.1021/acs.langmuir.5b03286

[b50] Sullan R. M. . Nanoscale structures and mechanics of barnacle cement. Biofouling 25, 263–275 (2009). 19180351 10.1080/08927010802688095

[b51] Dickinson G. H. . Barnacle cement: a polymerization model based on evolutionary concepts. J. Exp. Biol. 212, 3499–3510 (2009). 19837892 10.1242/jeb.029884 PMC2762877

[b52] Geurts P. . Synthetic spider silk fibers spun from pyriform spidroin 2, a glue silk protein discovered in orb-weaving spider attachment discs. Biomacromolecules 11, 3495–3503 (2010). 21053953 10.1021/bm101002w

[b53] Sponner A. . Characterization of the protein components of *Nephila clavipes* dragline silk. Biochemistry 44, 4727–4736 (2005). 15779899 10.1021/bi047671k

[b54] Vasanthavada K. . Spider glue proteins have distinct architectures compared with traditional spidroin family members. J. Biol. Chem. 287, 35986–35999 (2012). 22927444 10.1074/jbc.M112.399816 PMC3476267

[b55] Molliex A. . Phase separation by low complexity domains promotes stress granule assembly and drives pathological fibrillization. Cell 163, 123–133 (2015). 26406374 10.1016/j.cell.2015.09.015 PMC5149108

[b56] Yonemura N., Sehnal F., Mita K. & Tamura T. Protein composition of silk filaments spun under water by caddisfly larvae. Biomacromolecules 7, 3370–3378 (2006). 17154465 10.1021/bm060663u

[b57] Regier J. C. . Arthropod relationships revealed by phylogenomic analysis of nuclear protein-coding sequences. Nature 463, 1079–1083 (2010). 20147900 10.1038/nature08742

[b58] Blasingame E. . Pyriform spidroin 1, a novel member of the silk gene family that anchors dragline silk fibers in attachment discs of the black widow spider, *Latrodectus hesperus*. J. Biol. Chem. 284, 29097–29108 (2009). 19666476 10.1074/jbc.M109.021378 PMC2781455

[b59] Wolff J. O., Grawe I., Wirth M., Karstedt A. & Gorb S. N. Spider’s super-glue: thread anchors are composite adhesives with synergistic hierarchical organization. Soft Matter 11, 2394–2403 (2015). 25672841 10.1039/c4sm02130d

[b60] Walker G., Yule A. & Nott J. Structure and function in balanomorph larvae. Barnacle Biology. (Ed. Southward A. J.) 307–328 (A. A. Balkema, 1987).

[b61] Dougherty W. J. Barnacle adhesion - reattachment of the adult barnacle *Chthamalus-Fragilis Darwin* to polystyrene surfaces followed by centrifugational shearing. J. Crustacean Biol. 10, 469–478 (1990).

[b62] Burden D. K. . Barnacle *Balanus amphitrite* adheres by a stepwise cementing process. Langmuir 28, 13364–13372 (2012). 22721507 10.1021/la301695m

[b63] Gamo T., Inokuchi T. & Laufer H. Polypeptides of fibroin and sericin secreted from different sections of silk gland in *Bombyx mori. Insect* Biochemistry 7, 285–295 (1977).

[b64] Wang C. S., Ashton N. N., Weiss R. B. & Stewart R. J. Peroxinectin catalyzed dityrosine crosslinking in the adhesive underwater silk of a casemaker caddisfly larvae, Hysperophylax occidentalis. Insect Biochem. Mol. Biol. 54, 69–79 (2014). 25220661 10.1016/j.ibmb.2014.08.009

[b65] Flammang P., Lambert A., Bailly P. & Hennebert E. Polyphosphoprotein-Containing marine adhesives. J. Adhesion 85, 447–464 (2009).

[b66] Aldred N., Gohad N. V., Petrone L., Orihuela B., Liedberg B., Ederth T., Mount A., Rittschof D. & Clare A. S. Confocal microscopy-based goniometry of barnacle cyprid permanent adhesive. J. Exp. Biol. 216, 1969–1972 (2013). 23430996 10.1242/jeb.084913

[b67] Dickinson G. H., Yang X., Wu F. H., Orihuela B., Rittschof D. & Beniash E. Localization of Phosphoproteins within the Barnacle Adhesive Interface. Biol. Bull. 230, 233–242 (2016). 27365418 10.1086/BBLv230n3p233 PMC6377941

[b68] Maier G. P., Rapp M. V., Waite J. H., Israelachvili J. N. & Butler A. Adaptive synergy between catechol and lysine promotes wet adhesion by surface salt displacement. Science 349, 628–632 (2015). 26250681 10.1126/science.aab0556

[b69] Kyte J. & Doolittle R. F. A simple method for displaying the hydropathic character of a protein. J. Mol. Biol. 157, 105–132 (1982). 7108955 10.1016/0022-2836(82)90515-0

[b70] Ikai A. Thermostability and aliphatic index of globular proteins. J. Biochem. 88, 1895–1898 (1980). 7462208

[b71] Holm E. R., Orihuela B., Kavanagh C. J. & Rittschof D. Variation among families for characteristics of the adhesive plaque in the barnacle *Balanus amphitrite*. Biofouling 21, 121–126 (2005). 16167391 10.1080/08927010512331344188

[b72] Smith B. J. Protein sequencing protocols. In Methods in molecular biology (Ed. Smith B. J.) 123–131 (Humana Press, 2002).

[b73] Keller A., Nesvizhskii A. I., Kolker E. & Aebersold R. Empirical statistical model to estimate the accuracy of peptide identifications made by MS/MS and database search. Anal Chemistry 74, 5383–5392 (2002). 10.1021/ac025747h 12403597

[b74] Nesvizhskii A. I., Keller A., Kolker E. & Aebersold R. A statistical model for identifying proteins by tandem mass spectrometry. Anal. Chemistry 75, 4646–4658 (2003). 10.1021/ac0341261 14632076

[b75] Vizcaino J. A. . 2016 update of the PRIDE database and its related tools. Nucleic Acids Research 44, D447–D456 (2016). 26527722 10.1093/nar/gkv1145 PMC4702828

