# Supplementary material for: Sequence basis of Barnacle Cement Nanostructure is Defined by Proteins with Silk Homology
Source: Sci Rep. 2016 Nov 8;6:36219. doi: 10.1038/srep36219 (PMC5099703; doi:10.1038/srep36219)
Supplement: Supplementary Information [file srep36219-s1.pdf]

SUPPLEMENTARY INFORMATION SECTION

Sequence basis of Barnacle Cement Nanostructure is Defined by Proteins with Silk Homology

Christopher R. So[1,*], Kenan P. Fears[1], Dagmar H. Leary[2], Jenifer M. Scancella[1], Zheng Wang[2], Jinny Liu[2], Beatriz Orihuela[3], Dan Rittschof[3], Christopher M. Spillmann[2] and Kathryn J. Wahl[1,*]

[1]Chemistry Division, Code 6176, US Naval Research Laboratory, 4555 Overlook Ave, SW, Washington, DC, USA

[2]Center for Biomolecular Science and Engineering, Code 6900, US Naval Research Laboratory, 4555 Overlook Ave, SW, Washington, DC, USA

[3]Nicholas School of the Environment and Earth Sciences, Duke University Marine Laboratory, Beaufort, NC, USA

*Correspondence to: christopher.so@nrl.navy.mil or kathryn.wahl@nrl.navy.mil

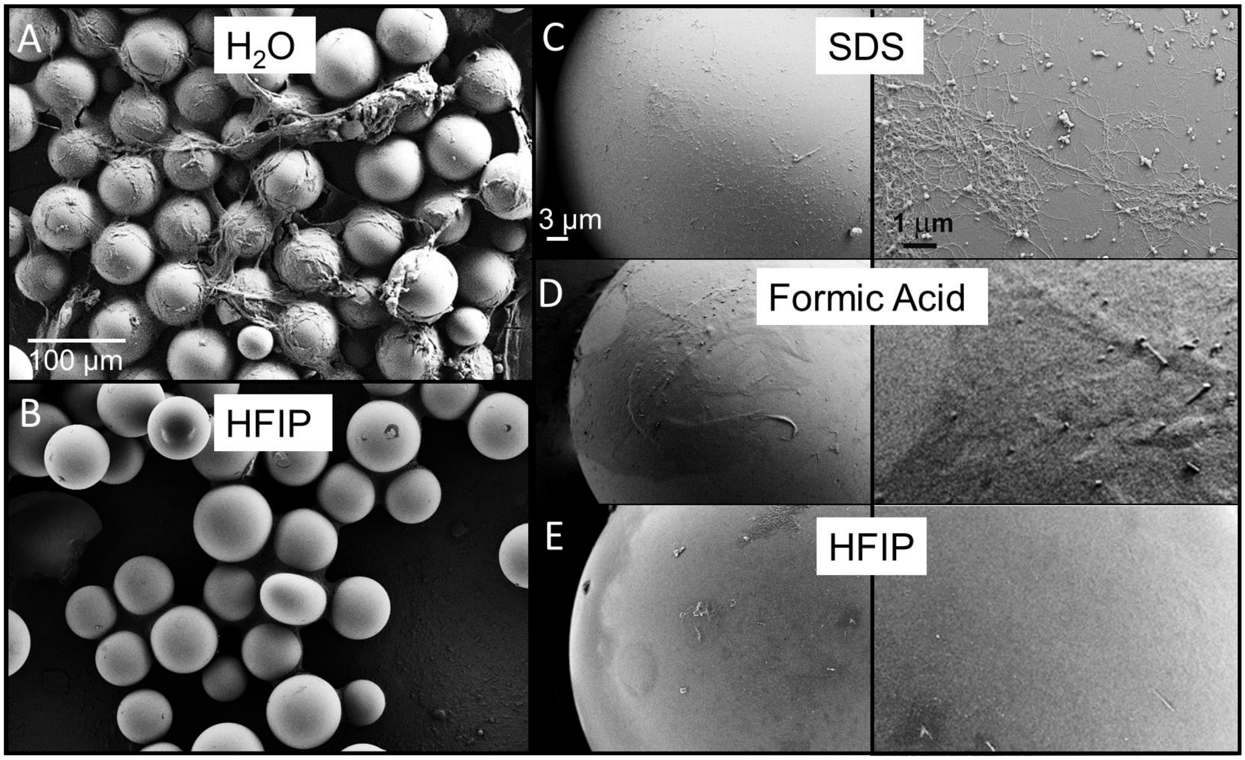

Supplementary Figure S1. Characterization of residual cement materials on beads after various solvent rinses, demonstrating effectiveness of HFIP. Barnacles were settled on beads for 3 days to produce a coated morphology observed in bead set A. Rinses were carried out by exposing beads to various solvents for 10 minutes under ambient temperatures and pressures. A) Beads attached to barnacle underside rinsed in artificial seawater, B) Beads rinsed in HFIP, magnified images of C) Sodium dodecyl sulfate (SDS), D) Formic Acid and E) HFIP. Scale bars represent images in each column.

Identified Proteins	Accession Number	Gummy 2015	Gummy 2016	Medallion 2016	Microsphere 2015	Microsphere 2016
NRLrnaseq,gb|AFY13482.1| MULTIFUNCin [Chthamalus fissus]	comp48163_c0_seq1_4	86	86	65	52	65
NRLrnaseq, ***** No hits found *****	comp41238_c0_seq1_4	18	29	13	21	32
Aacp100k-1	Aacp100k-1 (+1)	19	44	16	29	45
NRLrnaseq,|gb|AFY13480.1| MULTIFUNCin [Balanus glandula]	comp39924_c0_seq1_5	34	56	36	30	32
NRLrnaseq,ref|XP_001960248.1| uncharacterized protein Dana_GF11619,lysyl oxidase?[Drosophila ananassae]	comp44772_c0_seq1_4	13	20	18	12	19
Aacp114kDa	Aacp114kDa	2	31	8	22	37
Aacp19k	Aacp19k (+1)	12	12	7	6	10
NRLrnaseq,***** No hits found *****	comp47308_c0_seq1_4	10	20	1	20	25
NRLrnaseq,XP_013410251.1| PREDICTED: extracellular matrix protein FRAS1-like [Lingula anatina]	comp48220_c0_seq1_6	13	17	1	13	17
NRLrnaseq	comp40848_c1_seq1_4	1	12	0	16	15
NRLrnaseq,ref|XP_001946672.2| PREDICTED: chorion peroxidase-like [Acyrthosiphon pisum]	comp27704_c0_seq1_6	12	10	8	8	9
NRLrnaseq,***** No hits found *****	comp46881_c0_seq1_4	4	18	0	4	15
NRLrnaseq, ref|WP_007419168.1| hypothetical protein [Idiomarinasp. A28L]	comp39288_c0_seq1_5	6	23	3	9	16
NRLrnaseq,dbj|BAE94410.1| cement protein-19k, partial [Fistulobalanus albicostatus]	comp45928_c1_seq1_6	10	18	0	11	13
Shell,ref|XP_003292819.1hypotheticalproteinDICPUDRAFT_58166[Dictyosteliumpurpureum]	CL9487.Contig1_Ba_mix_	11	9	5	5	6
NRLrnaseq,gb|EFX80390.1| hypothetical protein DAPPUDRAFT_318553,peroxidase?, [Daphnia pulex]	comp45999_c0_seq1_4	10	7	8	6	7
NRLrnaseq,ref|XP_011183022.1| PREDICTED: antichymotrypsin-2-likeisoform X5 [Bactrocera cucurbitae]	comp45011_c0_seq1_5 (+	6	10	10	6	8
CYPRIDJPR2013,gb|AAR33079.1| settlement inducing protein complex [Amphibalanus amphitrite]	CL68.Contig2_Ba_mix (+1	1	9	18	0	23
NRLrnaseq,ref|XP_008945901.1| PREDICTED: LOW QUALITY PROTEIN: thimet oligopeptidase, partial [Merops nubicus]	comp135234_c0_seq1_5	7	4	3	4	4
NRLrnaseq,dbj|BAE94409.1| cement protein-19k [Megabalanus rosa]	comp40515_c0_seq1_5	7	9	1	5	6
NRLrnaseq,ref|XP_015373617.1| PREDICTED: peroxidase-like [Diuraphis noxia]	comp42709_c1_seq1_4	1	11	7	8	8
NRLrnaseq	comp45426_c2_seq1_2	2	5	4	1	4
NRLrnaseq,emb|CEP02176.1| hypothetical protein PBRA_002441[Plasmodiophora brassicae]	comp27343_c0_seq1_4	7	9	4	3	7
NRLrnaseq,ref|XP_011366188.1| PREDICTED: beta-1,4-N-acetylgalactosaminyltransferase 3 [Pteropus vampyrus]	comp38042_c0_seq1_4	1	5	5	4	6
NRLrnaseq,ref|WP_045875785.1| hypothetical protein [Frankiasp. DC12]	comp40644_c0_seq1_4	5	24	3	0	2
NRLrnaseq,emb|CBY30585.1| unnamed protein product [Oikopleura dioica]	comp42253_c0_seq1_4	4	11	1	2	7
NRLrnaseq, ***** No hits found *****	comp33562_c1_seq1_4	6	8	0	4	6
NRLrnaseq,ref|XP_006817093.1| PREDICTED: uncharacterized protein LOC100376421 [Saccoglossus kowalevskii]	comp47369_c0_seq1_5	1	11	3	0	0
NRLrnaseq,gb|KMQ91245.1| abc transporter permease [Lasius niger]	comp25334_c1_seq1_5	5	10	0	6	12
NRLrnaseq,dbj|BAM34601.1| waterborne settlement pheromone [Amphibalanus amphitrite]	comp25618_c0_seq1_5 (+	0	7	4	0	7
NRLrnaseq,ref|XP_014606419.1| PREDICTED: serine protease inhibitor 3/4-like isoform X6 [Polistes canadensis]	comp44898_c1_seq2_4	2	6	6	0	5
NRLrnaseq,ref|XP_012711402.1| PREDICTED: leukocyte elastase inhibitor-like isoform X2 [Fundulus heteroclitus]	comp42589_c2_seq1_5	5	10	3	4	6
NRLrnaseq, ***** No hits found *****	comp46137_c1_seq5_5	6	16	0	2	12
NP_034723.1 junction plakoglobin [Mus musculus]	28395018|ref	2	0	1	0	18
NRLrnaseq,ref|XP_013313762.1| hypothetical protein PV05_08770 [Exophiala xenobiotica]	comp42837_c0_seq1_6	2	10	3	2	1
NRLrnaseq,ref|XP_011871194.1| PREDICTED: venom allergen 3-like [Vollenhovia emeryi]	comp47983_c0_seq1_6	6	7	2	2	6
ACQ12582.1 Sequence 18 from patent US 7507710	228300741|gb (+2)	5	0	2	4	0
NRLrnaseq	comp27000_c0_seq1_6	0	6	1	0	2
NRLrnaseq, ref|XP_012246550.1| PREDICTED: papilin isoform X3 [Bombus impatiens]	comp46330_c0_seq1_6	5	7	5	9	8
NRLrnaseq,***** No hits found *****	comp27593_c0_seq1_5	6	9	1	3	6
NRLrnaseq, emb|CDZ62397.1| Two component sensor histidine kinaseFecR/PupR [Neorhizobium galegae bv. orientalis]	comp38220_c1_seq1_5	5	6	2	5	4
NRLrnaseq,ref|XP_009052071.1| hypothetical protein LOTGIDRAFT_159529 [Lottia gigantea]	comp32063_c0_seq1_4	4	7	3	2	3
NRLrnaseq,ref|XP_013775632.1| PREDICTED: peroxidase-like [Limulus polyphemus]	comp83572_c0_seq1_4	0	7	4	4	6
NRLrnaseq,gb|ACJ12892.1| vitellogenin 2 [Tigriopus japonicus]	comp47893_c0_seq1_2	0	1	2	0	13
NRLrnaseq,gb|AGR65306.1| hypothetical protein N134_07035 [Lactobacillusreuteri TD1]	comp43534_c0_seq1_4	5	5	0	3	2
NRLrnaseq	comp53583_c0_seq1_4	0	10	0	1	5
Shell,ref|XP_015373617.1PREDICTED:peroxidase-like[Diuraphisnoxia]	CL8390.Contig1_Ba_mix_4	0	0	20	0	0
NRLrnaseq,***** No hits found *****	comp56597_c0_seq1_4	5	5	4	3	2
NP_033739.1 actin, cytoplasmic 2 [Mus musculus]	6752954|ref	3	0	4	2	6
NRLrnaseq,ref|WP_035247368.1| phage host specificity protein [Actibacterium atlanticum]	comp55167_c0_seq1_4	3	6	2	2	1
NRLrnaseq	comp48743_c0_seq1_6	0	8	0	1	2
NRLrnaseq,gb|EFX81056.1| hypothetical protein DAPPUDRAFT_50513 lysyl oxidase?[Daphnia pulex]	comp43852_c1_seq1_2	3	3	4	3	2
Shell,gb|EFX74857.1hypotheticalproteinDAPPUDRAFT_323929(vitellogenin?)[Daphniapulex]	CL775.Contig1_Ba_mix_4	0	1	1	0	15
NRLrnaseq,ref|XP_006628687.1| PREDICTED: cell migration-inducing and hyaluronan-binding protein [Lepisosteus oculatus]	comp80448_c0_seq1_4	2	4	2	2	2
NRLrnaseq,ref|XP_013782980.1| PREDICTED: clotting factor B-like[Limulus polyphemus]	comp41724_c1_seq1_5	4	6	0	3	4
NRLrnaseq	comp44898_c1_seq4_5	0	3	3	1	7
NRLrnaseq	comp47216_c0_seq1_5 (+	0	0	4	0	7
NRLrnaseq,ref|XP_002072489.2| uncharacterized protein Dwil_GK12466 [Drosophila willistoni]	comp46953_c1_seq1_5 (+	0	1	2	0	9
NRLrnaseq	comp51115_c0_seq1_6	0	7	0	0	3
NRLrnaseq,ref|NP_001303466.1| uncharacterized protein Dmel_CG10407, isoform C [Drosophila melanogaster]	comp55944_c0_seq1_4	3	5	2	2	2
NP_001933.2 desmoglein-1 preproprotein [Homo sapiens]	119703744|ref	0	0	0	0	9
NRLrnaseq, ***** No hits found *****	comp46196_c0_seq1_5	1	3	0	1	6
NRLrnaseq,ref|XP_011265929.1| PREDICTED: uncharacterized protein LOC105257175 [Camponotus floridanus]	comp54786_c0_seq1_4	3	7	1	1	2
NRLrnaseq,ref|XP_014087846.1PREDICTED: serine protease snake-like [Bactrocera oleae]	comp25062_c0_seq1_5	4	7	0	0	1
NRLrnaseq,dbj|BAM34601.1| waterborne settlement pheromone [Amphibalanus amphitrite]	comp47419_c0_seq1_5 (+	0	0	2	0	5
Shell,gb|EGT46899.1hypotheticalproteinCAEBREN_29437[Caenorhabditisbrenneri]	Unigene4323_Ba_mix_1	2	4	1	4	0
NRLrnaseq	comp47727_c0_seq1_4	0	4	0	0	2
Shell,ref|XP_012283862.1PREDICTED:serineprotease42-likeisoformX2[Orussusabietinus]	CL200.Contig3_Ba_mix_3	0	0	1	0	8

NRLrnaseq	comp44495_c1_seq1_5	0	4	1	0	1
NRLrnaseq,ref|WP_045192707.1| bifunctional glutamine-synthetase adenylyltransferase/deadenyltransferase [Terrabacter sp.28]	comp48863_c0_seq1_5	2	4	0	0	3
NRLrnaseq,ref|XP_011067728.1| PREDICTED: peroxidase-like isoform X4 [Acromyrmex echinatior]	comp25540_c0_seq1_6	0	4	3	0	2
NRLrnaseq,ref|XP_014405601.1| PREDICTED: zonadhesin [Myotis brandtii]	comp43534_c0_seq2_4	6	5	1	0	0
NRLrnaseq,gb|AFY13482.1| MULTIFUNCin [Chthamalus fissus]	comp41028_c1_seq1_5	0	3	0	0	10
NRLrnaseq,***** No hits found *****	comp101276_c0_seq1_4	3	2	4	0	0
NRLrnaseq,ref|XP_013104691.1| PREDICTED: antichymotrypsin-2-like isoform X4 [Stomoxys calcitrans]	comp44997_c0_seq1_6	2	5	0	2	3
1H9Z|A Chain A, Human Serum Albumin Complexed With Myristic Acid And The R-(+) Enantiomer Of Warfarin	14719644|pdb (+2)	1	1	1	0	5
NRLrnaseq	comp55903_c0_seq1_6	0	4	0	0	1
NRLrnaseq,gb|KRG90602.1| hypothetical protein GLYMA_20G101900 [Glycine max]	comp47087_c0_seq1_6	0	0	0	1	4
NRLrnaseq	comp41421_c1_seq1_1	0	0	5	0	0
ADV40105.1 putative germinal histone H4 [Latrodectus hesperus]	318087026|gb (+15)	0	0	6	0	2
Shell,gb|EFX81683.1alpha-carbonicanhydrase[Daphniapulex]	CL15286.Contig1_Ba_mix_	0	0	5	0	0
NRLrnaseq	comp40607_c0_seq1_6	2	4	0	0	1
NRLrnaseq	comp40906_c1_seq1_5	1	5	0	0	1
P07355.2ANXA2_HUMAN RecName: Full=Annexin A2; AltName: Full=Annexin II; AltName: Full=Annexin-2; AltName: Full=Calpactin I heavy chain; AltName: Full=Calpactin-1 heavy chain; AltName: Full=Chromobindin-8; AltName: Full=Lipocortin II; AltName: Full=Placental anticoagulant protein IV; Short=PAP-IV; AltName: Full=Protein I; AltName: Full=p36	113950|sp (+8)	0	0	0	0	5
NRLrnaseq,ref|WP_015152096.1| DNA/RNA endonuclease G, NUC1 [Oscillatoria acuminata]	comp37235_c0_seq1_4	1	4	0	0	0
NP_077739.1 desmocollin-1 isoform Dsc1a preproprotein [Homo sapiens]	13435361|ref (+1)	0	0	0	0	5
NRLrnaseq	comp45721_c0_seq1_6 (+	0	0	0	0	4
NRLrnaseq	comp57366_c0_seq1_6	1	4	0	0	0
NRLrnaseq	comp63200_c0_seq1_4	4	2	0	0	0
NRLrnaseq	comp94854_c0_seq1_4	0	4	0	0	0
NRLrnaseq	comp81141_c0_seq1_1 (+	0	0	4	0	0

Supplementary Table S1. Unique peptide count determined by Scaffold software for whole cement samples prepared from various cement collection methods. Table shows a shared protein composition among all collection methods, where roughly half of the protein IDs share more than 4 peptides.

Identified Proteins	Accession Number	Coverslip	Gummy				Microsphere	
		NRL	NRLmod	NRLnomod	NRLTrypChymo	BioP	BioP	NRL
NRLrnaseq, ***** No hits found *****	comp41238_c0_seq1_4	9	12	14	14	12	14	18
NRLrnaseq,gb|AFY13482.1| MULTIFUNCin [Chthamalus fissus]	comp48163_c0_seq1_4	0	2	15	23	0	2	0
Aacp19k	Aacp19k (+1)	0	4	6	7	0	1	0
NRLrnaseq,***** No hits found *****	comp47308_c0_seq1_4	0	6	0	0	0	3	12
NRLrnaseq,dbj|BAE94409.1| cement protein-19k [Megabalanus rosa]	comp40515_c0_seq1_5	0	4	4	5	0	4	4
NRLrnaseq,ref|XP_014478061.1| PREDICTED: basement membrane-specific heparan sulfate proteoglycan core protein isoform X6 [Dinoponera quadriceps]	comp44361_c2_seq5_2 (+1)	20	0	0	0	0	0	0
NRLrnaseq,dbj|BAE94410.1| cement protein-19k, partial [Fistulobalanus albicostatus]	comp45928_c1_seq1_6	0	3	0	2	0	3	9
Aacp100k-1	Aacp100k-1 (+1)	1	0	1	0	0	1	8
NRLrnaseq,ref|XP_001960248.1| uncharacterized protein Dana_GF11619,lysyl oxidase?[Drosophila ananassae]	comp44772_c0_seq1_4	0	6	1	1	1	1	1
NRLrnaseq, ref|XP_012246550.1| PREDICTED: papilin isoform X3 [Bombus impatiens]	comp46330_c0_seq1_6	0	7	0	1	0	1	2
NRLrnaseq,ref|XP_002072489.2| uncharacterized protein Dwil_GK12466 [Drosophila willistoni]	comp46953_c1_seq1_5 (+1)	6	0	0	0	0	0	0
NRLrnaseq,ref|XP_012262935.1| PREDICTED: hemocytin [Athalia rosae]	comp81481_c0_seq1_6	8	0	0	0	0	0	0
NRLrnaseq,ref|XP_011640591.1| PREDICTED: basement membrane-specific heparan sulfate proteoglycan core protein isoform	comp43075_c0_seq1_4	9	0	0	0	0	0	0
CYPRIDJPR2013,ref|XP_012283862.1| PREDICTED: serine protease 42-like isoform X2 [Orussus abietinus]	CL200.Contig3_Ba_mix (+3)	4	0	0	0	0	0	0
Shell,ref|XP_002036473.1GM11791[Drosophilasechellia]	CL1641.Contig1_Ba_mix_5 (+2)	5	0	0	0	0	0	0

Supplementary Table S2. Number of unique peptides detected from band analysis of 63 kDa showing GSrCP-AA43-1 (protein ID comp41238_c0_seq1_4) to be the dominant protein across bands collected from various samples and digest conditions.

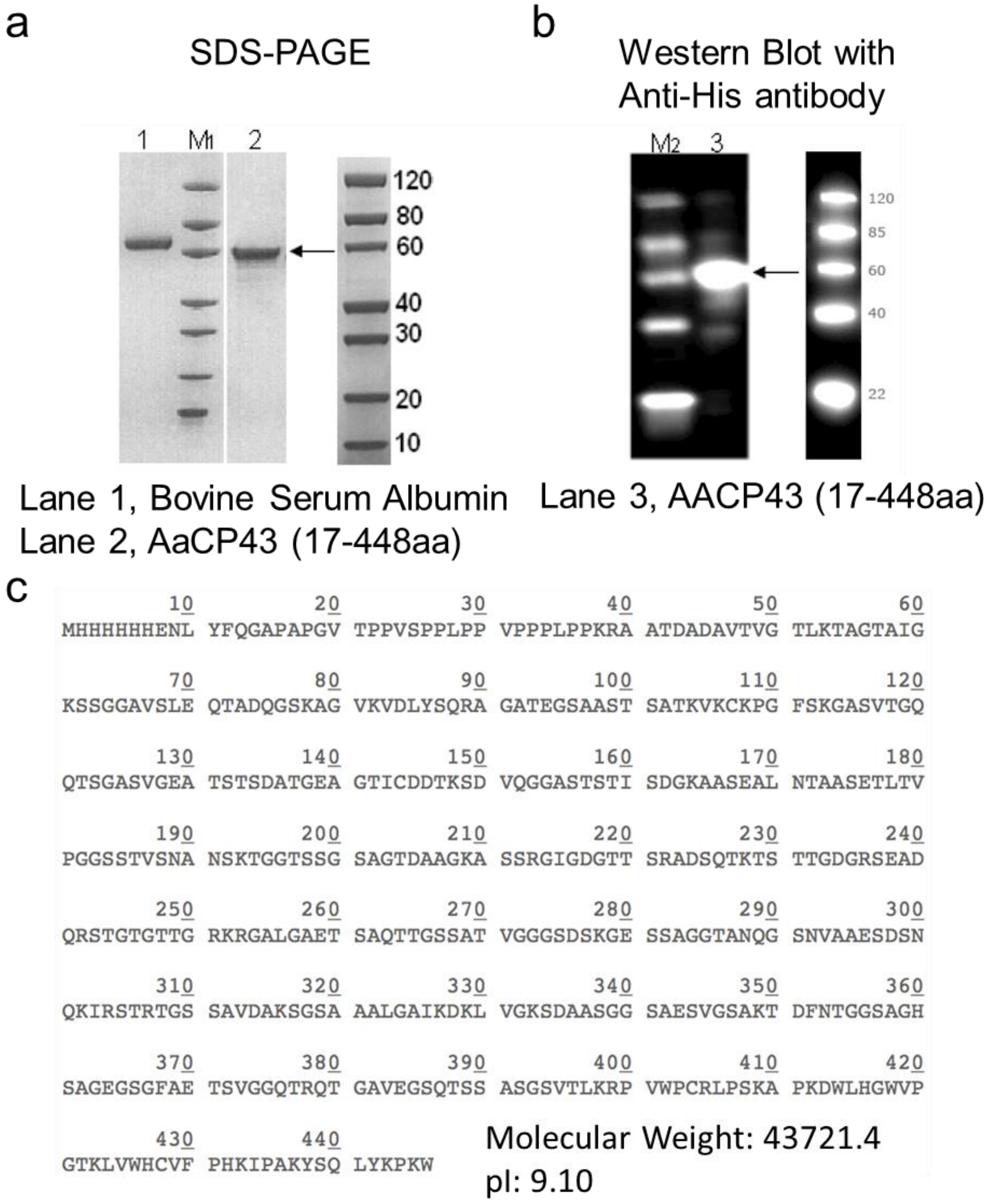

Supplementary Figure S2. Characterization of recombinant AaCP43 (17-448aa) protein. a) SDS-PAGE of purified His-AaCP43 protein running at 60 kDa, b) Western Blot of His-AaCP43 with anti-His antibody, and c) Full amino acid sequence of expressed product His-AaCP43.

Recombinant Protein Expression. Synthetic gene construct of the AaCP43 (17-448aa) coding region (Supplementary Figure S2) was designed and synthesized by Genscript (Piscataway, NJ) with a 6xHis tag as well as the removal of a 16 residue N-terminal transport sequence. The AaCP43 construct was subcloned into pET30a for expression in *E. coli* at Genscript facilities. *E. coli* strain BL21 (DE3) was transformed with the recombinant plasmid product. A single colony was then inoculated into LB medium containing antibiotics and cultures were incubated at 37 °C while shaking at 200 rpm overnight. IPTG was introduced into 1 L of TB culture when OD_{600} was between 0.5 and 1.0 to induce overexpression. Cells were harvested and pelleted by centrifugation. Cell pellets were lysed by sonication and inclusion bodies of insoluble AaCP43 were dissolved in 6M urea before purification. Pure AaCP43 was obtained using a two-step method; an affinity purification step on Ni resin was followed by gel filtration step on a Superdex 200 column. Fractions were pooled, refolded in PBS, and sterilized using a 0.22 μm filter. Proteins were analyzed by SDS-PAGE and Western blot using a mouse anti-His mAb (Genscript, A00186).

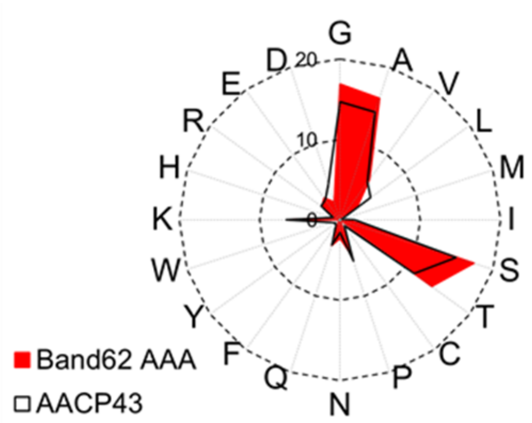

AA	Background pmol	Background %	62KD Band pmol	62KD Band %	Background Subtract pmol	Background Subtract %	AACP43 Transcript %
Asx	28.39	4.68	60.88	4.65	32.49	4.63	6.3
Ser	45.44	7.49	168.32	12.86	122.88	17.50	15.2
Glx	64.61	10.66	111.84	8.55	47.24	6.73	6.4
Gly	63.60	10.49	182.35	13.93	118.76	16.91	14.7
His	8.61	1.42	14.57	1.11	5.96	0.85	0.9
Arg	23.92	3.94	43.72	3.34	19.80	2.82	6.7
Thr	29.33	4.84	128.34	9.81	99.01	14.10	11.4
Ala	40.49	6.68	152.16	11.63	111.67	15.90	14.1
Pro	62.46	10.30	94.84	7.25	32.38	4.61	5.4
Cys	0.00	0.00	0.00	0.00	0.00	0.00	0.9
Tyr	94.89	15.65	93.17	7.12	-1.73	-0.25	0.7
Val	29.04	4.79	72.90	5.57	43.86	6.24	5.8
Met	7.77	1.28	5.48	0.42	-2.29	-0.33	0.2
Lys	30.62	5.05	75.28	5.75	44.67	6.36	6.7
Ile	20.36	3.36	27.08	2.07	6.72	0.96	1.8
Leu	40.50	6.68	58.73	4.49	18.22	2.59	4.7
Phe	16.28	2.68	19.02	1.45	2.74	0.39	0.9
Trp	0.00	0.00	0.00	0.00	0.00	0.00	1.1
Total	606.29	100	1308.66	100	702.37	100.00	100

Supplementary Figure S3. Amino acid analysis of 63 kDa PAGE band, showing high similarity with the theoretical composition of GSrCP-AA43-1 based on translated mRNA sequence. Experimental: Blank strip contained 13.7 ugs while 62kD protein strip contained 26.7 ugs as determined by hydrolysis and absorbance intensity of derivitized amino acids upon separation by HPLC.

```
   1  ATGCTGCCTGCCGCG ATCCTTCTCCTCTCC CTGGGTGCGGCGCTA TCGGCGCCTGCCCCC GGAGTGACGCCGCCC
   1   M  L  P  A  A   I  L  L  L  S   L  G  A  A  L   S  A  P  A  P   G  V  T  P  P
  76  GTTTCTCCACCTCTA CCGCCCGTTCCTCCA CCTCTTCCACCGAAG AGGGCTGCCACAGAC GCCGATGCGGTGACT
  26   V  S  P  P  L   P  P  V  P  P   P  L  P  P  K   R  A  A  T  D   A  D  A  V  T
 151  GTTGGTACGCTCAAG ACCGCAGGTACCGCC ATTGGTAAGTCCTCA GGTGGAGCTGTCTCT CTGGAGCAGACTGCC
  51   V  G  T  L  K   T  A  G  T  A   I  G  K  S  S   G  G  A  V  S   L  E  Q  T  A
 226  GATCAGGGAAGCAAA GCTGGAGTCAAGGTT GATCTCTACTCGCAG AGGGCGGGAGCTACG GAGGGAAGCGCCGCC
  76   D  Q  G  S  K   A  G  V  K  V   D  L  Y  S  Q   R  A  G  A  T   E  G  S  A  A
 301  TCCACCAGCGCGACC AAAGTCAAGTGCAAG CCTGGCTTCAGCAAG GGGGCGAGTGTGACC GGCCAGCAGACGAGC
 101   S  T  S  A  T   K  V  K  C  K   P  G  F  S  K   G  A  S  V  T   G  Q  Q  T  S
 376  GGCGCAAGTGTCGGC GAGGCGACGTCGACC TCCGACGCCACGGGA GAGGCGGGAACCATC TGCGACGATACCAAG
 126   G  A  S  V  G   E  A  T  S  T   S  D  A  T  G   E  A  G  T  I   C  D  D  T  K
 451  TCTGATGTCCAGGGA GGTGCCAGTACCTCC ACCATCAGCGACGGC AAGGCAGCTTCTGAG GCGCTCAACACCGCC
 151   S  D  V  Q  G   G  A  S  T  S   T  I  S  D  G   K  A  A  S  E   A  L  N  T  A
 526  GCCTCCGAAACGTTG ACGGTTCCCGGAGGA TCCAGCACAGTCTCC AATGCCAACTCGAAG ACCGGGGGTACCTCC
 176   A  S  E  T  L   T  V  P  G  G   S  S  T  V  S   N  A  N  S  K   T  G  G  T  S
 601  TCAGGATCCGCCGGA ACCGACGCAGCTGGC AAGGCTTCCAGTCGG GGAATCGGTGACGGC ACAACGTCACGTGCA
 201   S  G  S  A  G   T  D  A  A  G   K  A  S  S  R   G  I  G  D  G   T  T  S  R  A
 676  GATTCTCAAACGAAG ACATCGACGACAGGC GACGGAAGGAGCGAG GCTGACCAGCGCTCC ACCGGTACCGGAACC
 226   D  S  Q  T  K   T  S  T  T  G   D  G  R  S  E   A  D  Q  R  S   T  G  T  G  T
 751  ACAGGTCGCAAACGT GGGGCGCTCGGTGCC GAGACTTCTGCACAG ACCACGGGTAGTTCG GCAACCGTAGGAGGA
 251   T  G  R  K  R   G  A  L  G  A   E  T  S  A  Q   T  T  G  S  S   A  T  V  G  G
 826  GGATCAGATAGCAAG GGCGAGTCCTCCGCT GGAGGTACAGCCAAC CAGGGCTCCAACGTC GCCGCTGAGTCAGAC
 276   G  S  D  S  K   G  E  S  S  A   G  G  T  A  N   Q  G  S  N  V   A  A  E  S  D
 901  TCCAATCAAAAGATC AGATCAACGAGGACT GGTAGCTCCGCGGTG GATGCCAAGAGCGGC TCCGCTGCAGCGCTG
 301   S  N  Q  K  I   R  S  T  R  T   G  S  S  A  V   D  A  K  S  G   S  A  A  A  L
 976  GGTGCCATCAAAGAC AAACTCGTCGGAAAG AGTGACGCAGCTTCA GGAGGCAGCGCCGAG TCGGTCGGTAGCGCC
 326   G  A  I  K  D   K  L  V  G  K   S  D  A  A  S   G  G  S  A  E   S  V  G  S  A
1051  AAGACCGATTTCAAC ACCGGAGGTTCGGCT GGCCACTCGGCAGGG GAGGGCAGCGGTTTC GCCGAGACCTCTGTC
 351   K  T  D  F  N   T  G  G  S  A   G  H  S  A  G   E  G  S  G  F   A  E  T  S  V
1126  GGCGGTCAGACGCGT CAGACCGGTGCAGTC GAGGGAAGCCAGACA TCTAGCGCCAGCGGA AGCGTCACACTGAAA
 376   G  G  Q  T  R   Q  T  G  A  V   E  G  S  Q  T   S  S  A  S  G   S  V  T  L  K
1201  CGGCCTGTCTGGCCA TGCAGGCTCCCCAGC AAAGCGCCCAAAGAC TGGCTGCATGGCTGG GTGCCCGATACTAAA
 401   R  P  V  W  P   C  R  L  P  S   K  A  P  K  D   W  L  H  G  W   V  P  D  T  K
1276  TTGGTGTGGCCTTGT GTATTCCCTCACAAA ATACCCGCCAAGTAC AGCCAACTGTATAAG CCTAAATGGTAG
 426   L  V  W  P  C   V  F  P  H  K   I  P  A  K  Y   S  Q  L  Y  K   P  K  W  *
```

Supplementary Figure S4. GSrCP-AA43-1 RNA and translated amino acid sequences as verified by PCR.Three nucleotide mismatches between theoretical and mRNA are outlined in red.

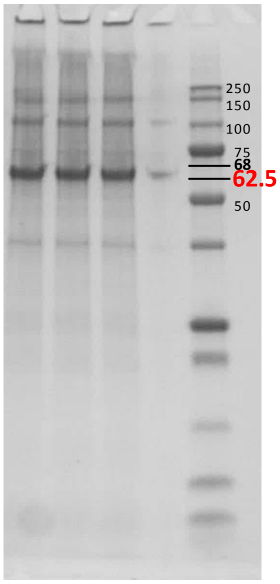

Microsphere											
PAGE MW	Facility	Enzyme	Transcript Entry #	Score	Transcript MW	pI	GRAVY	Aliphatic	CP Name	Sequence Homology	NCBI Annotation
100	BioP	mix	1::comp27593_c0_seq1_5	128	21877				43-like	43	
			1::comp56597_c0_seq1_4	103	31196						
			2::CL6240.Contig1_Ba_mix	102	60346						
			1::comp44772_c0_seq1_4	92	76364						Lysyl Oxidase
			1::comp33562_c0_seq1_3	88	16370				52-like	52	
			2::CL3592.Contig1_Ba_mix	82	41791						
			1::comp45928_c1_seq1_6	73	134253		-0.702	56.12	AACP48	19	
			1::comp27343_c0_seq1_4	69	28183				43-like	43	cross-beta structure silk protein 1
100	NRL	Tryp	1::comp41238_c0_seq1_4	642	62160		-0.423	56.14	AACP43	43	
			1::comp47308_c0_seq1_4	143	76040		-0.519	71.46	AACP57	57	
			AACP100k-1	57	114124				AACP100	100	
62.5	NRL	Tryp	1::comp41238_c0_seq1_4	1689	62160		-0.423	56.14	AACP43	43	
			1::comp47308_c0_seq1_4	384	76040		-0.519	71.46	AACP57	57	
			1::comp45928_c1_seq1_6	223	134253		-0.702	56.12	AACP48	19	
			1::comp40515_c0_seq1_5	220	70957		-0.387	67.65	AACP45	19	
			AACP100k-1	104	114124		0.178	117.08	AACP100	100	
			1::comp46330_c0_seq1_6	78	56017		-0.515	62.34	AACP38	19	
62.5	BioP	mix	1::comp41238_c0_seq1_4	1360	62160		-0.423	56.14	AACP43	43	
			1::comp47087_c0_seq1_6	841	9117						
			1::comp46330_c0_seq1_6	211	56017		-0.515	62.34	AACP38	19	
			2::Aacp19k	208	20154		0.013	92.17	AACP19	19	
			1::comp47308_c0_seq1_4	196	76040		-0.519	71.46	AACP57	57	
			1::comp45928_c1_seq1_6	153	134253		-0.702	56.12	AACP48	19	
			1::comp40515_c0_seq1_5	140	70957		-0.387	67.65	AACP45	19	
			1::comp44772_c0_seq1_4	130	76364						Lysyl Oxidase
			1::comp97185_c0_seq1_2	107	37285						

Supplementary Table S3. Band-based sequencing analysis of cement proteins from glass microspheres.

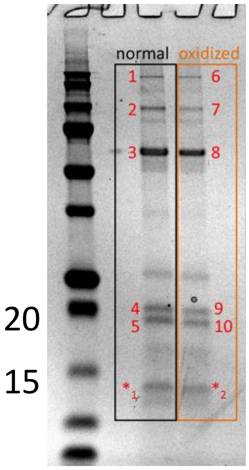

					Gummy						
PAGE MW	Facility	Enzyme	Transcript Entry #	Score	Transcript MW	pI	GRAVY	Aliphatic	CP Name	Sequence Homology	NCBI Annotation
250	NRL	Tryp	1::comp48163_c0_seq1_4	534	182231						SIPC
			1::comp101217_c0_seq1_1	139	19431						SIPC
			1::comp41238_c0_seq1_4	92	62160		-0.423	56.14	AACP43	43	
250	NRL	Tryp	1::comp48163_c0_seq1_4	518	182231						SIPC
62.5	NRL	Tryp	1::comp41238_c0_seq1_4	3109	62160		-0.423	56.14	AACP43	43	
			1::comp40515_c0_seq1_5	171	70957		-0.387	67.65	AACP45	19	
			1::comp46330_c0_seq1_6	69	56017		-0.515	62.34	AACP38	19	
			1::comp35623_c0_seq1_3	62	35040						
			1::comp47087_c0_seq1_6	60	9117						
62.5	NRL	Tryp	1::comp41238_c0_seq1_4	1575	62160		-0.423	56.14	AACP43	43	
			1::comp40515_c0_seq1_5	177	70957		-0.387	67.65	AACP45	19	
			1::comp44772_c1_seq1_2	89	16625						Neurotrypsin/ Serine Protease
62.5	BioP	mix	1::comp41238_c0_seq1_4	2884	62160		-0.423	56.14	AACP43	43	
			1::comp49909_c1_seq1_6	217	34116				43-like	43	
			1::comp39288_c0_seq1_5	158	114121						
			1::comp45426_c4_seq1_5	132	10903						Perlwapin
			1::comp27593_c0_seq1_5	128	21877				43-like	43	
			1::comp47308_c0_seq1_4	122	76040		-0.519	71.46	AACP57	57	
			1::comp45928_c1_seq1_6	86	134253		-0.702	56.12	AACP48	19	
			1::comp46137_c1_seq5_5	85	49470						
			1::comp45999_c0_seq1_4	81	62231						Chorion Peroxidase
			1::comp40515_c0_seq1_5	76	70957		-0.387	67.65	AACP45	19	
62.5	NRL	Tryp/Chym	1::comp41238_c0_seq1_4	496	62160		-0.423	56.14	AACP43	43	
			1::comp65098_c0_seq1_5	67	38746						
62.5	NRL	Tryp/Chym	1::comp41238_c0_seq1_4	564	62160		-0.423	56.14	AACP43	43	
20	NRL	Tryp	2::Aacp19k	572	20154		0.013	92.17	AACP19	19	
20	NRL	Tryp	2::Aacp19k	1443	20154		0.013	92.17	AACP19	19	
			1::comp148424_c0_seq1_1	65	9471						
20	NRL	Tryp	2::Aacp19k	227	20154		0.013	92.17	AACP19	19	
20	NRL	Tryp	2::Aacp19k	750	20154		0.013	92.17	AACP19	19	
14	NRL	Tryp	1::comp47369_c0_seq1_5	174	25169						Whey Acidic Protein
14	NRL	Tryp	1::comp65098_c0_seq1_5	55	38746						
14	NRL	Tryp/Chym	1::comp58019_c0_seq1_1	55	97596						
			1::comp129458_c0_seq1_5	53	8915						
14	NRL	Tryp/Chym	1::comp65098_c0_seq1_5	55	38746						

Supplementary Table S4. Band-based sequencing analysis of cement proteins from ‘opaque’ glue.

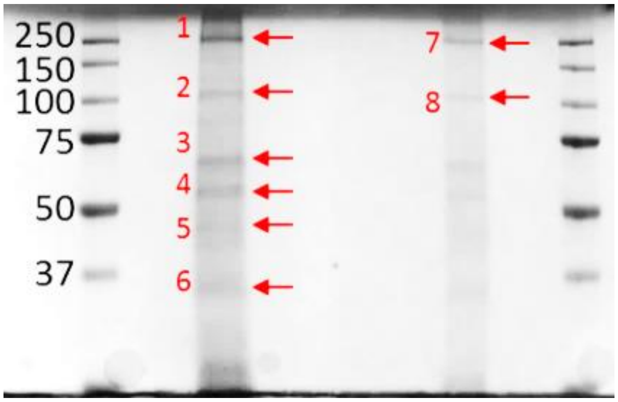

Figure for Supplementary Table S5. Bands excised for proteomic analysis.

Plaque

PAGE MW	Facility	Enzyme	Transcript Entry #	Database	Score	Transcript MW	CP Name	Sequence Homology
250	NRL	Tryp	1::comp48163_c0_seq1_4	barnacleRNAseqall	2381	182231		
			1::comp39924_c0_seq1_5	barnacleRNAseqall	348	227597		
			2::Aacp100k-1	barnNRLandDuke	231	114124	AACP100	100
			1::comp122409_c0_seq1_6	barnacleRNAseqall	65	9583		
			1::comp40644_c0_seq1_4	barnacleRNAseqall	65	174829	AACP105	105
			1::comp46070_c0_seq2_1	barnacleRNAseqall	64	86334		
			1::comp45011_c0_seq2_6	barnacleRNAseqall	60	63948		
250	NRL	Tryp	1::comp48163_c0_seq1_4	barnacleRNAseqall	1869	182231		
			1::comp39924_c0_seq1_5	barnacleRNAseqall	188	227597		
			2::Aacp100k-1	barnNRLandDuke	128	114124	AACP100	100
			1::comp30989_c1_seq1_3	barnacleRNAseqall	62	41834		
100	NRL	Tryp	1::comp25045_c1_seq1_2	barnacleRNAseqall	48	534529		
100	NRL	Tryp	1::comp40644_c0_seq1_4	barnacleRNAseqall	456	174829	AACP105	105
			1::comp45999_c0_seq1_4	barnacleRNAseqall	127	62231		
			2::Aacp100k-1	barnNRLandDuke	123	114124	AACP100	100
			1::comp84481_c0_seq1_4	barnacleRNAseqall	67	40630		
66.6	NRL	Tryp	1::comp45928_c1_seq1_6	barnacleRNAseqall	1035	134253	AACP48	19
			1::comp41238_c0_seq1_4	barnacleRNAseqall	588	62160	AACP43	43
			1::comp40644_c0_seq1_4	barnacleRNAseqall	230	174829	AACP105	105
			1::comp40515_c0_seq1_5	barnacleRNAseqall	140	70957	AACP45	19
			1::comp45011_c0_seq2_6	barnacleRNAseqall	92	63948		
			1::comp48163_c0_seq1_4	barnacleRNAseqall	91	182231		
			1::comp39288_c0_seq1_5	barnacleRNAseqall	91	114121		
			1::comp48863_c0_seq1_5	barnacleRNAseqall	90	17634		
			1::comp43534_c0_seq2_4	barnacleRNAseqall	88	54116		
			1::comp47983_c0_seq1_6	barnacleRNAseqall	88	52983		
			1::comp56597_c0_seq1_4	barnacleRNAseqall	75	31196		
			1::comp87868_c0_seq1_2	barnacleRNAseqall	71	9478		
			1::comp27343_c0_seq1_4	barnacleRNAseqall	65	28183	43-like	43
			1::comp44772_c0_seq1_4	barnacleRNAseqall	62	76364		
58.3	NRL	Tryp	1::comp41238_c0_seq1_4	barnacleRNAseqall	2742	62160	AACP43	43
			1::comp45928_c1_seq1_6	barnacleRNAseqall	221	134253	AACP48	19
			1::comp45011_c0_seq2_6	barnacleRNAseqall	174	63948		
			1::comp48220_c0_seq1_6	barnacleRNAseqall	147	54867	52-like	52
			1::comp40644_c0_seq1_4	barnacleRNAseqall	133	174829	AACP105	105
			1::comp44772_c0_seq1_4	barnacleRNAseqall	116	76364		
			1::comp27593_c0_seq1_5	barnacleRNAseqall	112	21877	43-like	43
			1::comp39288_c0_seq1_5	barnacleRNAseqall	103	114121		
			1::comp27704_c0_seq1_6	barnacleRNAseqall	89	53067		
			1::comp43534_c0_seq2_4	barnacleRNAseqall	77	54116		
			1::comp45999_c0_seq1_4	barnacleRNAseqall	67	62231		
			1::comp27343_c0_seq1_4	barnacleRNAseqall	65	28183	43-like	43
			1::comp49909_c1_seq1_6	barnacleRNAseqall	59	34116	43-like	43
43.5	NRL	Tryp	1::comp48163_c0_seq1_4	barnacleRNAseqall	1869	182231		
			1::comp39924_c0_seq1_5	barnacleRNAseqall	188	227597		
			2::Aacp100k-1	barnNRLandDuke	128	114124	AACP100	100
			1::comp30989_c1_seq1_3	barnacleRNAseqall	62	41834		
			1::comp84481_c0_seq1_4	barnacleRNAseqall	59	40630		
30	NRL	Tryp	1::comp40644_c0_seq1_4	barnacleRNAseqall	456	174829	AACP105	105
			1::comp45999_c0_seq1_4	barnacleRNAseqall	127	62231		
			2::Aacp100k-1	barnNRLandDuke	123	114124	AACP100	100
			1::comp84481_c0_seq1_4	barnacleRNAseqall	67	40630		

Supplementary Table S5. Band-based sequencing analysis of cement proteins from 3-day cover slip transfer.

amphitrite LOX vs. Drosophila melanogaster LOX

```
                           SRCR domain 1
Query  53   FTSLFGQRQFSVRLVGGSNSREGNVEVLIYGSRGVVGGWRSVCDIGWTTAHAEAVCRQLG  112
            +T +  + + ++RLVGG N  EGN+EVL  G       W +VCD  W +  A+ VCRQLG
Sbjct  50   YTKVLNKEEGAIRLVGGDNEYEGNIEVLHNGK------WGAVCDDEWDSTEADIVCRQLG  103

Query  113  FPGPAVATRHGRFGFRSTGTGTMVSNCQVGAGGRGLRDCFYRGVGTTEGSCNSDNVAGVI  172
            FPG    TR G FG         + N       + L DC + G G  E  C     AGV+
Sbjct  104  FPGMRRYTRSGFFG--PARRRFWMDNLFCEGHEQELVDCHFEGWG--ENDCEPGEAAGVV  159
                                             SRCR domain 2
Query  173  CGPD-----------------PSSPYGGR--MDVRLRGGGTR--GDVEVRYGGRGWGPVC  211
            C P                  P  P   R   + VRLRGG +R  G VEV   G  WG VC
Sbjct  160  CYPPENALIPMATPIIRDEDLPKYPIHSRSRLYVRLRGGRSRIEGRVEVSLDGGRWGSVC  219

Query  212  GDGFDVKDAMVVCRQLSLGAAKKSY---ISGRRASGEFILAGVECSGRETSLAQCRSERG  268
             DG+ + +A VVCRQL LG A +++     G       +L+G EC G ET LA C
Sbjct  220  ADGWSLLEANVVCRQLGLGYASEAFQTDFFGGFNVSRPVLSGSECYGNETELADCLHHDA  279

Query  269  DS--VRCAGNQFSGAAVECAGQQSRDLPDLRVDAQELQDSAILTTETLGDLECALDENCL  326
                + C GN+   AAV C        PDL VD  E++ +A L    +  ++CA++ENC+
Sbjct  280  SQGIISCHGNRQHVAAVIC----DYIAPDLVVDYLEIEQTAHLEDRPMLLMQCAMEENCV  335
                                                           Cu Binding
Query  327  AKTAAEIKRREPTLWRTRTRKLFRFTNKVWNNGKADYRPRADPSQWEWHACHEHYHSEES  386
            A  A +I+R +P  WR R+R+L +FT    N G AD+RP  + SQWEWH CH H+HS E
Sbjct  336  ANEAYQIQRDDPH-WRYRSRRLLKFTAAAINAGNADFRPFKEKSQWEWHMCHMHFHSMEV  394
                                Quinone co-factor linkage
Query  387  FSEYDLTYEGTDTKAAQGHKASFCLEDSECRRGITQKYQCYIERGTRPPQGIRAGCADIY  446
            F+ +D+ +     K AQGHKASFCLEDS C  G+ +KY C         QGI   C+D+Y
Sbjct  395  FATFDI-FNLRGIKVAQGHKASFCLEDSNCLPGVAKKYNC----ANSGDQGISINCSDVY  449
                                Cytokine receptor-like region
Query  447  GSYIDCQWIDVTDIRSGKYVLRVRINADRKVPEVSFDDNQVICSVDLNLEDETVRVTNCR  506
               +DCQW+DVTD+  G YVL++ IN + KV E+++D+N  IC  DL       RV NC+
Sbjct  450  LYNLDCQWVDVTDLIPGTYVLKIAINPEFKVAEMNYDNNAAIC--DLIYTANFARVQNCQ  507
```

SBJCT is Drosophila melanogaster

Supplementary Figure S5. Sequence alignment of *A. amphitrite* lysyl oxidase showing conserved substrate binding sites as DmLOXL-2 from *D. melanogaster* .

A. amphitrite Pxt vs. *H. occidentalis* Pxt

```
comp45999_c0_seq1_4 peroxinectin N-term, Sbjct is KM384736 H. occidentalis

Query  183  RVQARAASAAPRTTRPPRVTRPPPLLTSSLRRIGIPTL------PSFSPTNFRNVRAPRA  236
            ++++ AA      TT+ P  T+ P      S      +PT          + P    + +    +
Sbjct  25   KLKSTAAPVQKLTTKRPVTTKAPATTIKSPTVKSVPTTFVDTNSDEYDPIEAKKIYPFKT  84

Query  237  GLSIQGPVGVCVPEKPLNCNSGTPHRTYDGSCNNLYQTHWGRRLRGLRRLFDSTYWDSVY  296
               ++ P   C       C      +R++DGSCNNL  + WG         RL     Y D V
Sbjct  85   NEMLKPP---CCGRVIAACKKDDKYRSFDGSCNNLAHSDWGVPNATYSRLLPPVYSDGVR  141
                                                  Distal heme cavity
Query  297  SPRIHSVAGGLLPSARLVSAQVMSSSSVQHQLFTTAVTVFGQFADHDLSISPIFHRSGGY  356
             PR  S      LP AR V    +  +  V++  +    +  +GQF  HD+S+    F
Sbjct  142  LPR-RSTDKSPLPCARKVRTDLFPTGQVENGKWPLNIMYWGQFLAHDMSLLKDFD-----  195

Query  357  GSTSPIECCEPNHQFPAQPLHPQCFPIPVAANDPFYKTWNVRCHNFVRSIPAPDPQCL--  414
                 + CC  + Q+  +     C+PI  + A+DP       +RC NF R++    D  C
Sbjct  196  ---GLVSCCTADGQY-QKGNGRFCYPIKIPASDPTIGKAGIRCMNFTRTLTDKDMGCSVG  251
               Distal heme cavity
Query  415  PRPATQLNDITTWLDLSQVYGSERQQARGLRTSTGGRLKTST-GNLL--PRQPPPSGEC-  470
              PA QLN +T ++DLS VYG    + AR LRT T G +KT    GN +   P +P   +  C
Sbjct  252  NAPAKQLNTVTAYIDLSNVYGITDEIARSLRTLTNGEMKTEKRGNKVFPPSEPNKAANCP  311
                 Distal heme cavity
Query  471  ----VSGICFLAGDTRANENTLLTLLHTVLVREHNRVADRL  507
                   +C+  GD+R+N+N  L +    + +REHNR+A  L
Sbjct  312  CSTTSENVCYKTGDSRSNQNPQLAIQQIMFLREHNRLAKGL  352

comp27704_c0_seq1_6 peroxinectin C-term, Sbjct is KM384736 H. occidentalis
Query  3    VWLREHNRVAAALAARHTSWSDERLYQQARRINIAQWQHIVYREWLPTIVG-FQYANS--  59
            ++LREHNR+A  L + +  W+DERLYQ+ARRINIA +Q+I Y EWLP ++G    AN+
Sbjct  340  MFLREHNRLAKGLKSINPHWNDERLYQEARRINIAVFQYITYYEWLPILLGDSNLANNKI  399
                                Proximal heme cavity
Query  60   --VGLRGFGRYTYTPALRPDISTEFSTAAFR-LHSLVQGVISLVTQHGTLQRQLKLTENY  116
                + GF    Y     PDI+ E + AA+R  H+ + G++ L+       Q  + ++  +
Sbjct  400  IFPDIEGFVN-DYDSTKVPDITNEHAHAAYRQFHTQIAGMLQLMNSERQRQSTVPISNWF  458
                                                     Proximal heme cavity
Query  117  FRPDSLVITSLFTNMARGMTQQRPQSFDRRFVQAVRGDLFKFG--AFGLDLVAANIQRGR  174
             RP  L    +    +  GM  QR +  D  FV+ +    LF      FG+DL A +IQR R
Sbjct  459  NRPQILESGTNIEQLTVGMVTQRVEESDNIFVEQITDKLFACQGIGFGVDLKATDIQRDR  518

Query  175  DHALPTYATVAAACGSRSITGWFSFLSFMNWADVLRLKKVYAHWRDVDLFAGINLEKRAP  234
            DHAL  Y       CG +     W  +  F+  AD+ +LK +Y  ++DVD+  G  LE
Sbjct  519  DHALGYYNDYRKFCGLKVAQSWEDYGDFITPADIAKLKTLYKSYKDVDVSVGGGLEAIGE  578

Query  235  GAMVGPTARCVIADQFLRLRYGDRFFYDQAGQAGSFSWQQLQQLRRSSMARLLCDNVGAG  294
             A VGPT  C++ +QF R R  DRF+++        F+  QL+++R+ S +RL CDN G G
Sbjct  579  -AQVGPTFLCILNEQFRRTRQADRFWFENP--TSGFTLDQLREIRKGSSSRLFCDN-GDG  634

Query  295  FDSVQPLAFIRPIPLLNPVVGCQSYRIPKVDLS  327
               +QPL+F+    P  N  V C     I  +DL+
Sbjct  635  ITKIQPLSFVLIFPGFNQPVPCS--EITAMDLT  665
```

Supplementary Figure S6. Sequence alignment of two peroxinectin proteins from *A. amphitrite* showing conserved heme binding sites as *H. occidentalis*.

Rank	Description	Query Coverage	E value	Identity	Accession
	GSrCP-AA19-1				
1	19 kDa cement protein [Amphibalanus amphitrite]	1	7.00E-141	1	AKZ20819.1
2	cement protein-19k [Fistulobalanus albicostatus]	0.85	2.00E-77	0.68	BAE94410.1
3	cement protein-19k [Balanus improvisus]	0.85	3.00E-60	0.55	BAE94411.1
4	cement protein-19k [Megabalanus rosa]	0.98	2.00E-59	0.51	BAE94409.1
	AACP20-1				
1	cement protein 20 kDa-1 [Amphibalanus amphitrite]	0.92	7.00E-87	0.98	AFX74689.1
2	cement protein 20 kDa-3 [Amphibalanus amphitrite]	0.84	1.00E-40	0.59	AFX74691.1
3	cement protein 20 kDa-2 [Amphibalanus amphitrite]	0.91	2.00E-27	0.36	AFX74690.1
4	20kDa-cement protein homologue [Fistulobalanus albicostatus]	0.86	7.00E-26	0.35	BAF96022.1
5	cement protein-20k [Megabalanus rosa]	0.93	1.00E-10	0.27	BAB18762.1
	AACP20-2				
1	cement protein 20 kDa-2 [Amphibalanus amphitrite]	1	1.00E-106	0.91	AFX74690.1
2	20kDa-cement protein homologue [Fistulobalanus albicostatus]	0.96	3.00E-46	0.48	BAF96022.1
3	cement protein 20 kDa-1 [Amphibalanus amphitrite]	0.98	3.00E-33	0.37	AFX74689.1
4	cement protein 20 kDa-3 [Amphibalanus amphitrite]	0.71	4.00E-21	0.36	AFX74691.1
5	cement protein-20k [Megabalanus rosa]	0.77	7.00E-16	0.3	BAB18762.1
	LrCP-AA52-1				
1	52 kDa cement protein [Amphibalanus amphitrite]	1	0	0.99	AKZ20820.1
2	52kDa cement protein [Megabalanus rosa]	0.99	2.00E-158	0.46	BAL22342.1
3	[Megabalanus rosa=acorn barnacles, secondary cement, insoluble fraction, Peptide Partial, 28 aa]	0.08	3.00E-05	0.61	AAB46749.1
	LrCP-AA100-2				
1	114 kDa cement protein [Amphibalanus amphitrite]	1	0	0.99	AKZ20818.1
2	cement protein 100k [Amphibalanus amphitrite]	0.98	0	0.58	AGS19349.1
3	cement protein-100k [Megabalanus rosa]	1	0	0.44	BAB12269.1
	LrCP-AA100-1				
1	cement protein 100k [Amphibalanus amphitrite]	1	0	1	AGS19349.1
2	114 kDa cement protein [Amphibalanus amphitrite]	0.84	0	0.58	AKZ20818.1
3	cement protein-100k [Megabalanus rosa]	0.84	0	0.49	BAB12269.1
	GSrCP-AA19-2				
1	cross-beta structure silk protein 1 [Mallada signata]	0.98	9.00E-32	0.28	ACN87361.1
2	silk sericin MG-1 [Galleria mellonella]	0.92	7.00E-25	0.25	AGN03940.1
3	cross-beta structure silk protein 2 [Mallada signata]	0.91	8.00E-25	0.27	ACN87362.1
4	fibroin 2 [Dolomedes tenebrosus]	0.94	2.00E-23	0.28	AAK30599.1
5	heavy-chain filboin, putative [Pediculus humanus corporis]	0.94	2.00E-22	0.28	XP_002432277.1
6	dragline silk protein [Nephila clavipes]	0.95	3.00E-22	0.28	AAL32375.1
7	efibroin [Aposthonia gurneyi]	0.82	2.00E-21	0.25	ABW24185.1
8	minor ampullate spidroin 1-like protein [Latrodectus hesperus]	0.93	3.00E-21	0.26	ACB29694.1
9	minor ampullate spidroin-like protein [Nephilengys cruentata]	0.9	3.00E-21	0.26	ABR37276.1
10	spidroin 1 [Nephila clavipes]	0.92	6.00E-21	0.29	AAC38957.1
11	major ampullate spidroin-like protein [Latrodectus geometricus]	0.9	2.00E-20	0.25	AAV91960.1
12	cement protein-19k [Fistulobalanus albicostatus]	0.86	2.00E-20	0.36	BAE94410.1
	GSrCP-AA43-1				
1	cross-beta structure silk protein 1 [Mallada signata]	0.8	3.00E-33	0.3	ACN87361.1
2	silk sericin MG-1 [Galleria mellonella]	0.81	7.00E-29	0.26	AGN03940.1
3	cross-beta structure silk protein 2 [Mallada signata]	0.8	3.00E-26	0.28	ACN87362.1
4	silk sericin MG-2 [Galleria mellonella]	0.78	5.00E-25	0.22	AGN03941.1
5	efibroin [Aposthonia gurneyi]	0.78	2.00E-24	0.26	ABW24185.1
6	mucin related 29B [Drosophila melanogaster]	0.8	5.00E-24	0.25	NP_723377.1
7	silk fibroin [Haploembia solieri]	0.8	8.00E-23	0.27	AEC12432.1
8	sericin 1 [Bombyx mandarina]	0.82	2.00E-22	0.27	ADY69178.1
9	sericin 1A' [Bombyx mori]	0.81	3.00E-22	0.26	BAD00699.1
10	serine-rich protein3 [Samia ricini]	0.86	5.00E-22	0.23	BAS31055.1
11	sericin 3 precursor [Bombyx mori]	0.81	1.00E-21	0.23	NP_001108116.1
12	serine-rich protein5 [Samia ricini]	0.77	1.00E-21	0.24	BAS31057.1
	GSrCP-AA19-5				
1	cross-beta structure silk protein 1 [Mallada signata]	0.95	1.00E-10	0.25	ACN87361.1
2	sericin 3 precursor [Bombyx mori]	0.88	1.00E-09	0.21	NP_001108116.1
3	cross-beta structure silk protein 2 [Mallada signata]	0.94	1.00E-09	0.22	ACN87362.1
4	serine-rich protein3 [Samia ricini]	0.85	6.00E-09	0.22	BAS31055.1

5	hornet silk protein Vssilk 4 [Vespa simillima xanthoptera]	0.92	1.00E-08	0.21	BAJ09449.1
6	hornet silk protein Vssilk 4 [Vespa simillima xanthoptera]	0.92	1.00E-08	0.21	BAF95004.1
7	sericin 1 precursor [Bombyx mori]	0.86	2.00E-08	0.22	NP_001037506.1
8	fibroin 1 [Euagrus chisoseus]	0.95	3.00E-08	0.22	ABW80568.1
9	sericin 1 [Bombyx mandarina]	0.85	3.00E-08	0.22	ADY69178.1
10	heavy chain fibroin [Hepialus californicus]	0.91	3.00E-08	0.24	ADE58103.1
11	cement protein-19k [Fistulobalanus albicostatus]	0.57	4.00E-08	0.24	BAE94410.1
12	silk sericin MG-1 [Galleria mellonella]	0.93	4.00E-08	0.2	AGN03940.1
	GSrCP-AA19-4				
1	cement protein-19k [Fistulobalanus albicostatus]	0.69	1.00E-28	0.38	BAE94410.1
2	19 kDa cement protein [Amphibalanus amphitrite]	0.81	2.00E-22	0.33	AKZ20819.1
3	GL10349 [Drosophila persimilis]	0.16	2.00E-22	0.54	XP_002016723.1
4	Diacylglycerol kinase kappa [Melipona quadrifasciata]	0.22	3.00E-21	0.4	KOX74686.1
5	RecName: Full=Protein TsetseEP; AltName: Full=EP-repeat protein; Flags: Precursor [Glossina morsitans morsitans]	0.15	3.00E-21	0.54	Q95P09.1
6	RecName: Full=Protein TsetseEP; AltName: Full=EP-repeat protein; Flags: Precursor [Glossina palpalis palpalis]	0.15	6.00E-21	0.54	Q8T4N5.1
7	cement protein-19k [Megabalanus rosa]	0.81	3.00E-20	0.32	BAE94409.1
8	cement protein-19k [Balanus improvisus]	0.7	8.00E-20	0.34	BAE94411.1
9	hypothetical protein DAPPUDRAFT_98962 [Daphnia pulex]	0.35	1.00E-17	0.32	EFX85380.1
10	silk sericin MG-1 [Galleria mellonella]	0.7	2.00E-17	0.24	AGN03940.1
11	cross-beta structure silk protein 1 [Mallada signata]	0.72	1.00E-16	0.27	ACN87361.1
12	Cell surface glycoprotein 1 [Papilio xuthus]	0.31	2.00E-16	0.44	KPI98596.1
	GSrCP-AA19-3				
1	cement protein-19k [Megabalanus rosa]	0.81	4.00E-29	0.35	BAE94409.1
2	cement protein-19k [Fistulobalanus albicostatus]	0.74	1.00E-27	0.4	BAE94410.1
3	19 kDa cement protein [Amphibalanus amphitrite]	0.84	3.00E-25	0.34	AKZ20819.1
4	cement protein-19k [Balanus improvisus]	0.74	1.00E-21	0.35	BAE94411.1
5	RecName: Full=Protein TsetseEP; AltName: Full=EP-repeat protein; Flags: Precursor [Glossina morsitans morsitans]	0.18	1.00E-16	0.49	Q95P09.1
6	RecName: Full=Protein TsetseEP; AltName: Full=EP-repeat protein; Flags: Precursor [Glossina palpalis palpalis]	0.16	2.00E-16	0.55	Q8T4N5.1
7	GL10349 [Drosophila persimilis]	0.22	3.00E-16	0.38	XP_002016723.1
8	Diacylglycerol kinase kappa [Melipona quadrifasciata]	0.38	3.00E-16	0.51	KOX74686.1
9	pyriform spidroin 2 [Nephila clavipes]	0.93	5.00E-15	0.24	ADK92884.1
10	Poly(U)-specific endoribonuclease like protein [Melipona quadrifasciata]	0.71	1.00E-13	0.25	KOX78239.1
11	hypothetical protein DAPPUDRAFT_98962 [Daphnia pulex]	0.31	4.00E-13	0.36	EFX85380.1
12	GL19041 [Drosophila persimilis]	0.18	3.00E-12	0.43	XP_002014267.1
	comp27593_c0_seq1_5				
1	cross-beta structure silk protein 1 [Mallada signata]	0.94	2.00E-11	0.32	ACN87361.1
2	major ampullate spidroin 1 [Kukulcania hibernalis]	0.94	9.00E-11	0.3	AAT08433.1
3	egg case silk protein 2 [Latrodectus hesperus]	0.72	2.00E-10	0.33	ABC68105.1
4	cross-beta structure silk protein 2 [Mallada signata]	0.89	3.00E-10	0.32	ACN87362.1
5	major ampullate spidroin [Agelenopsis aperta]	0.72	3.00E-10	0.35	ADM14325.1
6	efibroin [Aposthonia gurneyi]	0.76	3.00E-10	0.29	ABW24185.1
7	major ampullate spidroin [Agelenopsis aperta]	0.72	6.00E-10	0.35	AAT08436.1
8	MiSp [Uloborus diversus]	0.97	7.00E-10	0.32	ABD61597.1
9	silk fibroin [Antipaluria urichi]	0.72	8.00E-10	0.3	ACJ04053.1
10	lingerer, isoform L [Drosophila melanogaster]	0.77	1.00E-09	0.32	NP_001303338.1
11	lingerer, isoform K [Drosophila melanogaster]	0.77	1.00E-09	0.32	NP_001260795.1
12	lingerer, isoform J [Drosophila melanogaster]	0.77	3.00E-09	0.32	NP_001260794.1

Supplementary Table S6. Top 12 unfiltered nrNCBI search results for GSrCP and LrCP proteins.

Right to Left	Top to bottom
SIPC	Aacp19k 203 aa
comp55944_c0_seq1_4	comp45928_c1_seq1_6,
Aacp19k 203 aa	comp40515_c0_seq1_5,
comp45928_c1_seq1_6,	comp46137_c1_seq5_5
comp40515_c0_seq1_5,	comp46330_c0_seq1_6,
comp46137_c1_seq5_5	comp170921_c0_seq1_1
comp46330_c0_seq1_6	comp87199_c0_seq1_5
comp33102_c0_seq1_4	comp33102_c0_seq1_4
comp88850_c0_seq1_5	comp88850_c0_seq1_5
comp170921_c0_seq1_1	comp83572_c0_seq1_4
comp87199_c0_seq1_5	comp25062_c0_seq1_5
comp56597_c0_seq1_4	comp44772_c0_seq1_4,
comp43534_c0_seq1_4	comp41238_c0_seq1_4,
comp43852_c1_seq1_2	comp49909_c1_seq1_6,
comp44898_c1_seq1_4	comp27593_c0_seq1_5,
comp44997_c0_seq1_6	comp27343_c0_seq1_4
comp45011_c0_seq1_5	comp41724_c1_seq1_5
comp79394_c0_seq1_2	comp45426_c4_seq1_5,
CL1249.Contig1_Ba_mi	comp43852_c1_seq1_2
comp43534_c0_seq2_4	comp81853_c0_seq1_6
comp60205_c0_seq1_5	Aacp100k(HK)
comp44772_c0_seq1_4,	comp84577_c0_seq1_4
comp41724_c1_seq1_5	comp135234_c0_seq1_5
comp81853_c0_seq1_6	comp25334_c1_seq1_5
comp45426_c4_seq1_5,	comp47308_c0_seq1_4,
comp25062_c0_seq1_5	comp46196_c0_seq1_5
comp83572_c0_seq1_4	Aacp52k
comp41238_c0_seq1_4,	comp33562_c1_seq1_4
comp56597_c0_seq1_4,	comp33562_c0_seq1_3
comp27593_c0_seq1_5,	comp48220_c0_seq1_6
comp27343_c0_seq1_4	comp48863_c0_seq1_5
comp49909_c1_seq1_6	Aacp20k-2 136 aa
Aacp20k-1	comp39288_c0_seq1_5
comp33562_c1_seq1_4	comp43534_c0_seq1_4
comp33562_c0_seq1_3	comp47983_c0_seq1_6
Aacp52k	comp43534_c0_seq2_4
comp48220_c0_seq1_6	comp80764_c0_seq1_4
Aacp114k-1 (NRL)	CL215.Contig2_Ba_mix
comp48863_c0_seq1_5	comp80527_c0_seq1_6
Aacp20k-2 136 aa	comp44898_c1_seq1_4
comp47308_c0_seq1_4,	comp45011_c0_seq1_5
comp46196_c0_seq1_5	comp44997_c0_seq1_6
comp25334_c1_seq1_5	CL1249.Contig1_Ba_mi
comp47983_c0_seq1_6	comp79394_c0_seq1_2
comp84577_c0_seq1_4	Aacp20k-1
comp135234_c0_seq1_5	comp60205_c0_seq1_5
comp39288_c0_seq1_5	SIPC
Aacp100k(HK)	comp55944_c0_seq1_4
comp80764_c0_seq1_4	Aacp114k-1 (NRL)
CL215.Contig2_Ba_mix	comp56597 c0 seq1 4,
comp80527_c0_seq1_6	

Homologous Pairs	
X	Y
comp45928_c1_seq1_6	Comp27343_c0_seq1_4
Comp25062_c0_seq1_5	Comp41724_c1_seq1_5
AACP114 (NRL)	AACP100 (HK)
AACP20-1	AACP20-2
Comp43534_c0_seq1_4	Comp43534_c0_seq2_4
Comp80764_c0_seq1_4	Comp55944_c0_seq1_4
AACP100 (HK)	AACP114 (NRL)
AACP20-2	AACP20-1
Comp41724_c1_seq1_5	Comp25062_c0_seq1_5

Supplementary Tables S7 and S8. Corresponding protein IDs for 2D heat map in Figure 2 and discrete pairs.

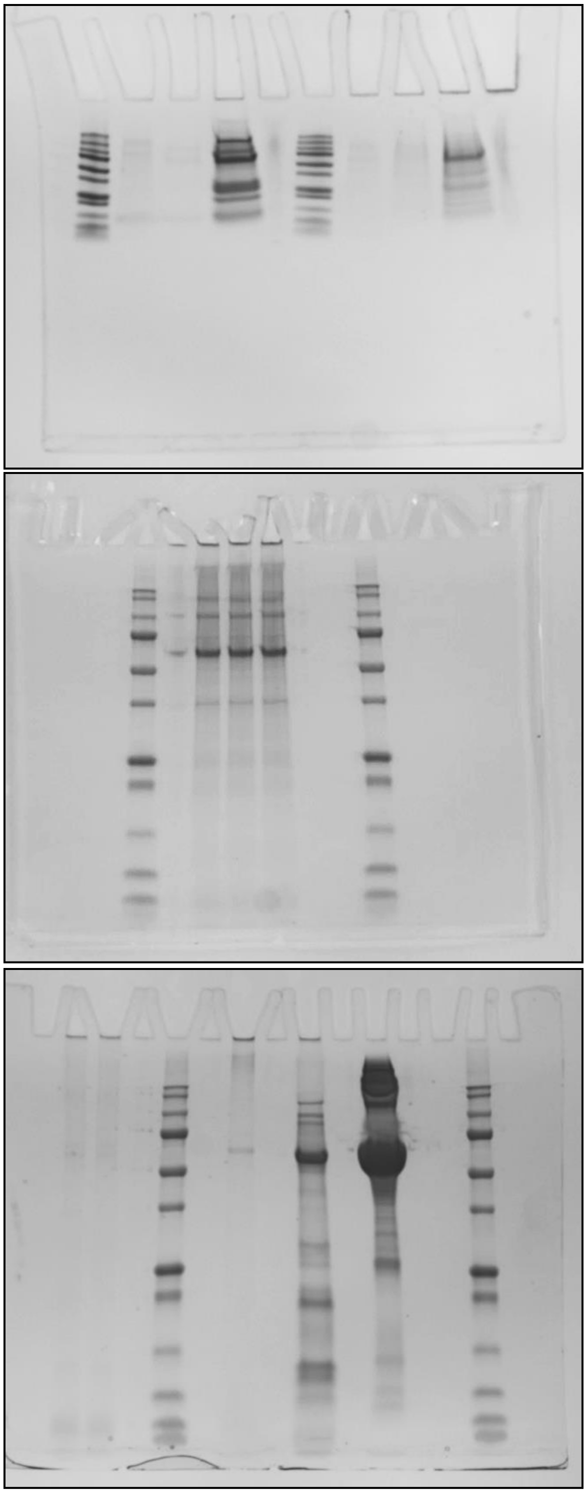

Supplementary Figure S7. Uncropped whole gel images from Figure 1: top- solubility of cement as shown in Fig. 1b from the main text, middle- cement from microsphere as shown in Fig. 1c, and bottom, cement from opaque glue as shown in Fig. 1c.
